# Zinc–gold cooperative catalysis for the direct alkynylation of benzofurans

**DOI:** 10.3762/bjoc.9.204

**Published:** 2013-08-29

**Authors:** Yifan Li, Jérôme Waser

**Affiliations:** 1 Laboratory of Catalysis and Organic Synthesis, Ecole Polytechnique Fédérale de Lausanne, EPFL SB ISIC LCSO, BCH4306, 1015 Lausanne, Switzerland

**Keywords:** alkynylation, benzofurans, cooperative catalysis, direct functionalization, gold catalysis, hypervalent iodine

## Abstract

The direct alkynylation of benzofurans was achieved for the first time using the hypervalent iodine reagent 1-[(triisopropylsilyl)ethynyl]-1,2-benziodoxol-3(1*H*)-one (TIPS-EBX) based on the cooperative effect between a gold catalyst and a zinc Lewis acid. High selectivity was observed for C2-alkynylation of benzofurans substituted with alkyl, aryl, halogen and ether groups. The reaction was also successful in the case of the more complex drug 8-methoxypsoralen (8-MOP).

## Introduction

**Figure 1:**
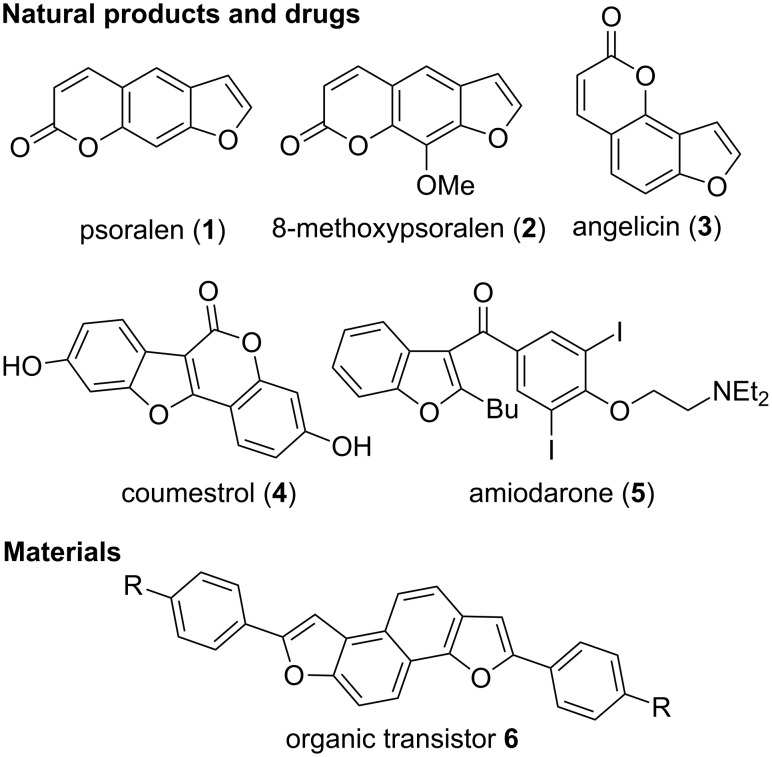
Benzofurans in bioactive compounds and materials.

Benzofurans are important heterocycles frequently encountered in both bioactive compounds and organic materials (Figure 1). For example, members of the furocoumarin class of natural products including psoralen (**1**), 8-methoxypsoralen (**2**) and angelicin (**3**) can cross-link with DNA upon light irradiation. They have consequently been used for the treatment of skin diseases such as cancer or psoriasis [1–4]. The natural product coumestrol (**4**) is found especially in soy beans and has estrogenic activity [5]. Synthetic bioactive compounds containing benzofurans are also important, as exemplified by amiodarone (**5**), as antiarrythmic drug [6–7]. Finally, benzofurans have also emerged recently as important structural elements for organic materials, such as the organic transistor **6** [8].

Due to the importance of benzofurans, the discovery of new efficient methods for their synthesis and functionalization is an intensive field of research [9–11]. Especially interesting would be methods allowing the direct and regioselective C–H functionalization of benzofurans [12]. In this context, the introduction of an alkyne would be particularly useful, as acetylenes are important building blocks in synthetic chemistry, chemical biology and materials science [13]. Nevertheless, to the best of our knowledge, the direct alkynylation of benzofurans is still an unknown process.

**Scheme 1:**
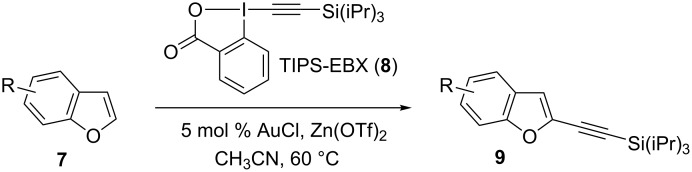
Zinc–gold catalyzed C2-alkynylation of benzofurans.

Since 2009, our group has developed a mild gold-catalyzed [14–17] method for the alkynylation of electron-rich aryls such as indoles and pyrroles [18], thiophenes [19], anilines [20] and furans [21]. Key for success was the use of ethynylbenziodoxolones, which are cyclic hypervalent iodine reagents [22–23]. Nevertheless, the conditions we have used for other heterocycles gave only very low yields in the case of benzofurans. Herein, we would like to report the first catalytic direct C2-alkynylation of benzofurans **7** based on a cooperative effect between a gold catalyst and a zinc Lewis acid using 1-[(triisopropylsilyl)ethynyl]-1,2-benziodoxol-3(1*H*)-one (TIPS-EBX, **8**) as reagent (Scheme 1). The reaction proceeded under mild conditions (60 °C under air) and could also be used to alkynylate the more complex polycyclic natural product 8-methoxypsoralen (**2**).

## Findings

Benzofuran (**7a**) is less reactive then furans and indeed no product was observed under the conditions optimized for the latter [21] at room temperature or at 60 °C using the commercially available electrophilic alkynylation reagent TIPS-EBX (**8**) (Table 1, entries 1 and 2) [24–27]. Fortunately, benzofuran (**7a**) was also more stable in the presence of acidic additives, and co-activation became possible, whereas Zn(OTf)_2_ was superior to trifluoroacetic acid (TFA) at 60 °C (Table 1, entries 3 and 4) [19,28]. No product was observed in the absence of AuCl, demonstrating the cooperative effect of the two metals (Table 1, entry 5). Lower or higher temperatures did not increase the yield (Table 1, entries 6 and 7). Finally, using a larger excess of TIPS-EBX (**8**) and Zn(OTf)_2_ gave 75% yield of alkynylation product **9a** (Table 1, entry 8). The use of Zn(OTf)_2_ in catalytic amount led to a lower yield (Table 1, entry 9), and a larger excess resulted in decomposition of the starting material only (Table 1, entry 10). Although other Lewis acids could also be used (Table 1, entries 11 and 12) [29], no better results than with Zn(OTf)_2_ were obtained (Table 1, entry 7). Importantly, in contrast to our previous work with benzothiophenes [19], high selectivity for C2 alkynylation was observed.

**Table 1:** Optimization of the alkynylation of benzofuran (**7a**).

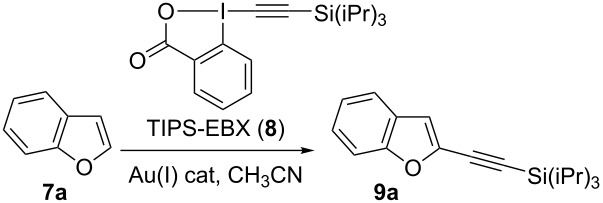				

Entry^a^	Equiv **8**	Additive^b^	*T* [°C]	Yield

**1**	1.2	**–**	23	<5%
**2**	1.2	**–**	60	<5%
**3**	1.2	TFA	60	42%
**4**	1.2	Zn(OTf)_2_	60	56%
**5**	1.2	Zn(OTf)_2_^c^	60	<5%
**6**	1.2	Zn(OTf)_2_	40	48%
**7**	1.2	Zn(OTf)_2_	82	36%
**8**	2	Zn(OTf)_2_	60	75%
**9**	2	Zn(OTf)_2_^d^	60	37%
**10**	2	Zn(OTf)_2_^e^	60	0%
**11**	2	Zn(NTf)_2_	60	57%
**12**	2	Yb(OTf)_3_	60	62%

^a^Reaction conditions: **7a** (0.20 mmol) and AuCl (0.01 mmol) in acetonitrile (0.8 mL) under air for 26 h, isolated yield; ^b^same amount as **8**; ^c^without gold catalyst; ^d^0.2 equiv; ^e^4.0 equiv.

**Scheme 2:**
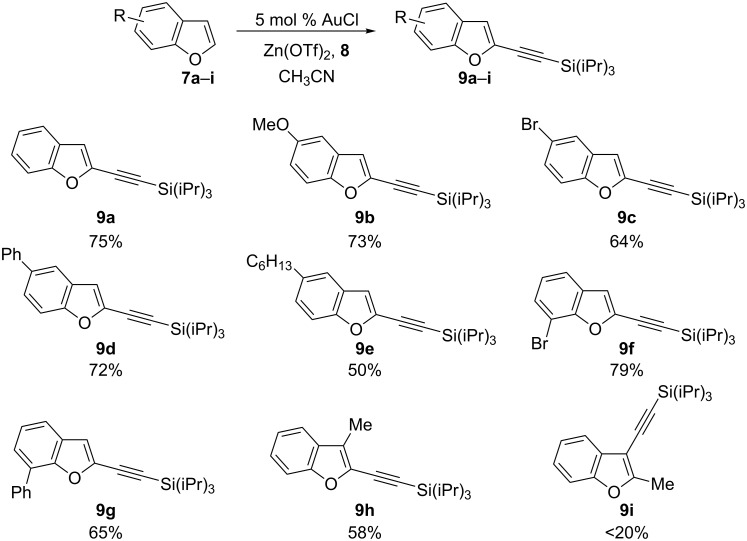
Scope of the reaction.

**Scheme 3:**
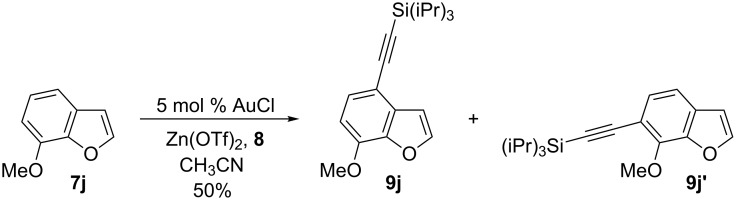
Alkynylation of 7-methoxybenzofuran (**7j**) [31].

The scope of the reaction was then investigated (Scheme 2). Substitution by diverse functional groups was first examined on the C5 position. An electron-rich methoxy group was well tolerated, giving the desired alkynylation product **9b** in 73% yield. The reaction was also successful with a bromide substituent (product **9c**), making the method orthogonal to classical cross-coupling chemistry [30]. In presence of an aryl or an alkyl substituent, alkynylation was also obtained in 72% and 50% respectively (products **9d** and **9e**). Benzofurans substituted at the C7 position could also be used, as demonstrated by the efficient formation of alkynes **9f** and **9g**. In contrast, when 7-methoxybenzofuran (**7j**) was used, no C2 alkynylation product could be isolated. Instead, a mixture of C4 and C6 alkynylated benzofurans **9j** and **9j**’ was obtained (Scheme 3) [31]. Substitution on the furan ring was also possible at the C3 position (product **9h**), but the use of 2-methylbenzofuran (**7i**) led to very a low yield in the alkynylation reaction.

**Scheme 4:**
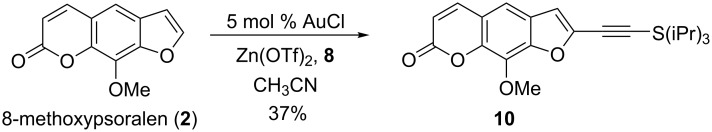
Alkynylation of 8-methoxypsoralen (**2**).

Finally, we wondered if the alkynylation method could also be successful in the case of more complex benzofuran-containing natural products and drugs. We were pleased to see that the alkynylation of 8-methoxypsoralen (**2**) was indeed possible. The major product **10** bearing the acetylene group at the C5’ position was obtained in 37% yield (Scheme 4) [32]. Although the yield was still moderate, this was one of the first examples of direct alkynylation of a marketed drug. It also gave access in a single step to an interesting furocoumarin derivative with an extended chromophore, which could be important for phototherapy.

Mechanistically, the reaction could proceed either via π-activation of the triple bond by the gold catalyst followed by conjugate addition of the benzofuran, α-elimination and 1,2-shift, or oxidative addition of TIPS-EBX (**8**) onto the gold catalyst (either at the Au(I) or Au(0) oxidation level) followed by electrophilic auration and reductive elimination [33]. The role of the zinc Lewis acid is not completely clear at this stage, but it may act by complexing the carboxylate group of the hypervalent iodine reagent, enhancing its electrophilic reactivity [19,34]. In fact, a complete shift of the ^1^H NMR signals of TIPS-EBX (**8**) was observed when Zn(OTf)_2_ was added, whereas no signal shift was observed when mixing the Lewis acid and benzofuran (**7a**) [35].

In conclusion, the first direct alkynylation method of benzofurans has been developed. Key for success was a cooperative effect between a gold catalyst and a zinc Lewis acid, together with the use of the hypervalent iodine reagent TIPS-EBX (**8**). Preliminary results obtained with 8-methoxypsoralen (**2**) demonstrated that the reaction could also be applied to more complex furocoumarin natural products.

## Experimental

**General procedure for the alkynylation of benzofurans:** TIPS-EBX (**8**, 342 mg, 0.800 mmol, 2.0 equiv), AuCl (4.6 mg, 0.020 mmol, 0.050 equiv), Zn(OTf)_2_ (289 mg, 0.800 mmol, 2.0 equiv) and benzofuran **7** (0.40 mmol, 1.0 equiv) were added into CH_3_CN (2.0 mL) under air. The mixture was stirred for 26 hours at 60 °C. Then the mixture was concentrated in presence of silica gel and purified directly by column chromatography.

## Supporting Information

File 1 Experimental part.
