# Supplementary material for: Zinc–gold cooperative catalysis for the direct alkynylation of benzofurans
Source: Beilstein J Org Chem. 2013 Aug 29;9:1763–7. doi: 10.3762/bjoc.9.204 (PMC3778369; doi:10.3762/bjoc.9.204)
Supplement: File 1 — Experimental part. [file Beilstein_J_Org_Chem-09-1763-s001.pdf]

Supporting information

for

Zinc–gold cooperative catalysis for the direct alkynylation of benzofurans

Yifan Li[1] and Jérôme Waser*[1]

Address: [1] Laboratory of Catalysis and Organic Synthesis, Ecole Polytechnique Fédérale de Lausanne, EPFL SB ISIC LCSO, BCH4306, 1015 Lausanne, Switzerland

Email: Jérôme Waser - jerome.waser@epfl.ch

* Corresponding author

Experimental part

Table of contents

1. General method s3
2. Preparation of reagents s4
3. Synthesis of starting material. s4
4. Alkynylation reactions s6
5. Spectra of new compounds..s15

1. General methods

All reactions were carried out in oven dried glassware under an atmosphere of nitrogen, unless stated otherwise. For quantitative flash chromatography technical grade solvents were used. For flash chromatography for analysis, HPLC grade solvents from Sigma-Aldrich were used. THF, Et_2O, CH_3CN, toluene, hexane and CH_2Cl_2 were dried by passage over activated alumina under nitrogen atmosphere (H_2O content < 10 ppm, *Karl-Fischer* titration). NEt_3 and pyridine were distilled under nitrogen from KOH. Gold chloride was purchased from Aldrich and kept in a desiccator under anhydrous conditions (decrease of reactivity has been observed for the catalyst if prolonged exposition to air (ca 1 month). The solvents were degassed by the Freeze-Pump-Thaw method when mentioned. All chemicals were purchased from Acros, Aldrich, Fluka, VWR, Aplichem or Merck and used as such unless stated otherwise. Chromatographic purification was performed as flash chromatography using Macherey-Nagel silica 40–63, 60 Å, using the solvents indicated as eluent with 0.1–0.5 bar pressure. TLC was performed on Merck silica gel 60 F_{254} TLC glass plates or aluminium plates and visualized with UV light, permanganate stain, CAN stain or anisaldehyde stain. Melting points were measured on a Büchi B-540 melting point apparatus using open glass capillaries, the data are uncorrected. ^{1}H NMR spectra were recorded on a Bruker DPX-400 400 MHz spectrometer in chloroform-*d*, DMSO-d_6 or CD_3OD, all signals are reported in ppm with the internal chloroform signal at 7.26 ppm, the internal DMSO signal at 2.50 ppm or the internal methanol signal at 3.30 ppm as standard. The data is being reported as (s = singlet, d = doublet, t = triplet, q = quadruplet, qi = quintet, m = multiplet or unresolved, br = broad signal, app = apparent, coupling constant(s) in Hz, integration, interpretation).^{13}C NMR spectra were recorded with ^{1}H-decoupling on a Bruker DPX-400 100 MHz spectrometer in chloroform-*d*, DMSO-d_6 or CD_3OD, all signals are reported in ppm with the internal chloroform signal at 77.0 ppm, the internal DMSO signal at 39.5 ppm or the internal methanol signal at 49.0 ppm as standard. Infrared spectra were recorded on a JASCO FTIR B4100 spectrophotometer with an ATR PRO410-S and a ZnSe prisma and are reported as cm^{-1} (w = weak, m = medium, s = strong, br = broad). Gas chromatographic and low resolution mass spectrometric measurements were performed on a Perkin-Elmer Clarus 600 gas chromatograph and mass spectrometer using a Perkin-Elemer Elite fused silica column (length: 30 m, diameter: 0.32 mm) and helium as carrier gas. High resolution mass spectrometric measurements were performed by the mass spectrometry service of ISIC at the EPFL on a MICROMASS (ESI) Q-TOF Ultima API. HPLC measurement were done on a JASCO HPLC system with an AS2055 Autosampler, a PU 2089 Pump, a UV 2075 detector and a SEDEX 85 (SEDERE) detector using a CHIRALPAK IC column from DAICEL Chemical Industries Ltd. HPLC grade solvents from Sigma-Aldrich were used.

2. Preparation of reagents

TIPS-EBX is commercially available, or can be synthesized using a reported procedure.[1]

3. Synthesis of starting materials.

5-Phenylbenzofuran (7d)

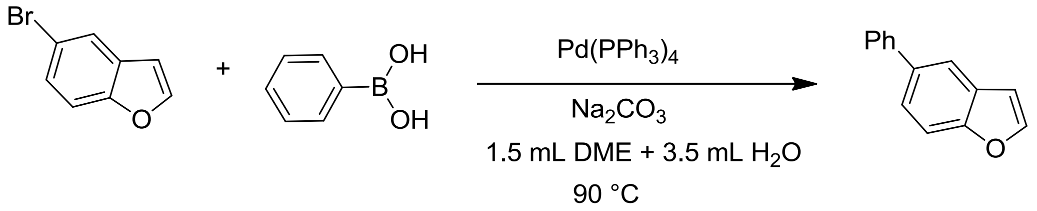

7c **15** **7d**

A schlenk tube was charged with boronic acid **15** (152 mg, 1.25 mmol, 1.25 equiv), $Pd(PPh_3)_4$ (57.5 mg, 0.0500 mmol, 0.05 equiv), Na_2CO_3 (265 mg, 2.50 mmol, 2.50 equiv), DME (1.5 mL) and H_2O (3.5 mL). After degassing the mixture, 5-bromobenzofuran (**7c**) (196 mg, 1.00 mmol, 1.0 equiv) was added. The mixture was heated to 90 °C and stirred overnight under N_2. Then, the mixture was filtered through a plug of celite, and diluted with CH_2Cl_2 (10 mL). The organic layer was washed with 1 M NaOH (10 mL), brine (10 mL), dried over $MgSO_4$ and concentrated under reduced pressure. The crude product was purified by column chromatography (SiO_2; pentane) to afford **7d** as a white solid (174 mg, 0.900 mmol, 90%).

R_f (pentane): 0.6.
^{1}H NMR (400 MHz, $CDCl_3$) δ 7.82 (d, 1 H, *J* = 1.4 Hz; *FuranH*), 7.68 (d, 1 H, *J* = 2.2 Hz; *ArH*), 7.65 (dd, 2 H, *J* = 8.5, 1.3 Hz, *ArH*), 7.60 (d, 1 H, *J* = 8.5 Hz; *ArH*), 7.56 (dd, 1 H, *J* = 8.6, 1.3 Hz, *ArH*), 7.48 (t, 2 H, *J* = 7.4 Hz, *ArH*), 7.38 (t, 1H, *J* = 7.4 Hz; *ArH*) 6.84 (d, 1 H, *J* = 1.4 Hz; *FuranH*) ^{13}C NMR (101 MHz, $CDCl_3$) δ 154.5, 145.6, 141.6, 136.4, 128.7, 127.9, 127.4, 126.8, 123.9, 119.7, 111.5, 106.8. The NMR data correspond to the literature.[2]

5-Hexylbenzofuran (7e)

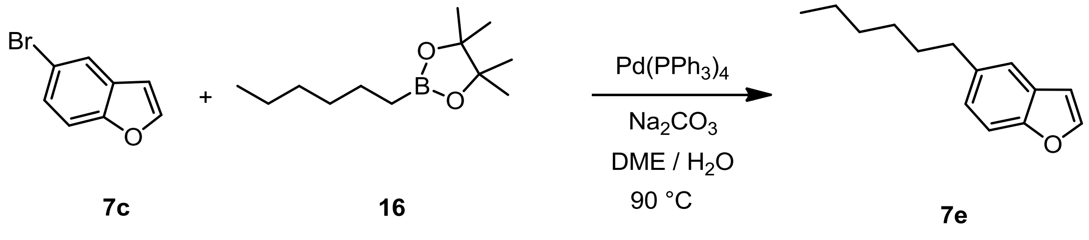

7c **16** **7e**

[1] J. P. Brand, J. Waser, *Synthesis* **2012**, *44*, 1155.
[2] Varela-Fernandez, A.; Gonzalez-Rodriguez, C.; Varela, J.; Castedo, L.; Saa, C. *Org. Lett.* **2009**, *11*, 5350.

A schlenk tube was charged with boronic ester **16** (170 mg, 0.800 mmol, 1.0 equiv), $Pd(PPh_3)_4$ (46.2 mg, 0.0500 mmol, 0.05 equiv), Na_2CO_3 (85 mg, 2.5 mmol, 1.0 equiv), DME (1.0 mL) and H_2O (2.5 mL). After degassing the mixture, 5-bromobenzofuran (**7c**, 173 mg, 0.880 mmol, 1.1 equiv) was added. The mixture was heated to 90 °C and stirred overnight under N_2. Then, the mixture was filtered through a plug of celite, and diluted with CH_2Cl_2 (10 mL). The organic layer was washed with 1 M NaOH (10 mL), brine (10 mL), dried over $MgSO_4$ and concentrated under reduced pressure. The crude product was purified by column chromatography (SiO_2; pentane) to afford **7e** as a colorless oil (77 mg, 0.38 mmol, 48%).

R_f (pentane): 0.5.
^{1}H NMR (400 MHz, $CDCl_3$) δ 7.59 (d, 1 H, J = 2.2 Hz, *FuranH*), 7.41 (m, 2 H, *ArH*), 7.12 (dd, 1 H, J = 8.4, 1.7 Hz, *ArH*), 6.72 (dd, 1 H, J = 2.2, 0.8 Hz, *FuranH*), 2.70 (m, 2 H, *HexylH*), 1.65 (m, 2 H, *HexylH*), 1.40-1.26 (m, 6 H, *HexylH*), 0.90 (t, 3 H, J = 7.0 Hz, *HexylH*). ^{13}C NMR (101 MHz, $CDCl_3$) δ 153.4, 144.9, 137.4, 127.4, 124.9, 120.3, 110.8, 106.4, 35.9, 32.1, 31.7, 29.0, 22.6, 14.1. IR 2956 (m), 2928 (s), 2857 (m), 2362 (w), 1697 (w), 1468 (s), 1331 (w), 1263 (m), 1197 (w), 1130 (m), 1113 (w), 1034 (w), 910 (w), 885 (w), 810 (m), 764 (m), 737 (s). Anal. calcd. for $C_{14}H_{18}O$: C, 83.12%; H, 8.97%. Found: C, 83.24%; H, 8.89%.

7-Phenylbenzofuran (7g)

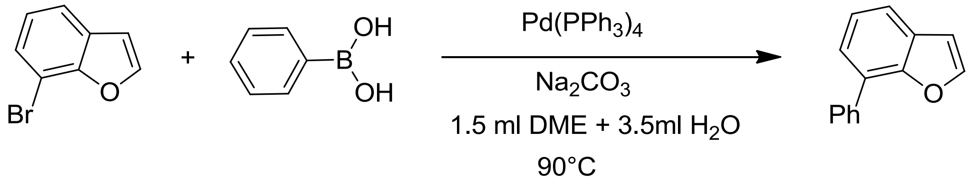

7f **15** **7g**

A schlenk tube was charged with boronic acid **15** (89 mg, 0.73 mmol, 1.0 equiv), $Pd(PPh_3)_4$ (42 mg, 0.050 mmol, 0.05 equiv), Na_2CO_3 (77 mg, 0.73 mmol, 1.0 equiv), DME (1.0 mL) and H_2O (2.5 mL). After degassing the mixture, 7-bromobenzofuran (**7f**, 158 mg, 0.880 mmol, 1.1 equiv) was added. The mixture was heated to 90 °C and stirred overnight under N_2. Then, the mixture was filtered through a plug of celite, and diluted with CH_2Cl_2 (10 mL). The organic layer was washed with 1 M NaOH (10 mL), brine (10 mL), dried over $MgSO_4$ and concentrated under reduced pressure. The crude product was purified by column chromatography (SiO_2; pentane) to afford **7g** as a colorless oil (126 mg, 0.649 mmol, 89%).

R_f (pentane): 0.3.
^{1}H NMR (400 MHz, $CDCl_3$) δ 7.92 (m, 2 H, *ArH*), 7.72 (d, 1 H, J = 2.2 Hz, *FuranH*), 7.63 (dd, 1 H, J = 7.7, 1.1 Hz, *ArH*), 7.55 (m, 2 H, *ArH*), 7.51 (dd, 1 H, J = 7.5, 1.1 Hz, *ArH*), 7.44 (m, 1 H, *ArH*), 7.37 (m, 1 H, *ArH*), 6.87 (d, 1 H, J = 2.2 Hz, *FuranH*). ^{13}C NMR (101 MHz, $CDCl_3$) δ 152.2, 145.0, 136.5, 128.6, 128.5, 128.1, 127.6, 125.6, 123.8, 123.3, 120.4, 106.7. The NMR data corresponds to the literature.[3]

[3] Otterlo, W.; Morgans, G.; Lee, M.; Kuzvidza, S.; Moleele, S.; Thornton, N.; Koning, C. T, Teth, *Tetrahedron,* **2005** , *61*, 7746.

4. Alkynylation Reactions

Optimization of the reaction:

General procedure:

TIPS-EBX (**8**), AuCl (2.3 mg, 0.010 mmol, 0.050 equiv) and additives were added into a solution of benzofuran (**7a**, 23.6 mg 0.200 mmol, 1.0 equiv) in CH_3CN under air. Then the mixture was concentrated with silica gel and purified by column chromatography directly.

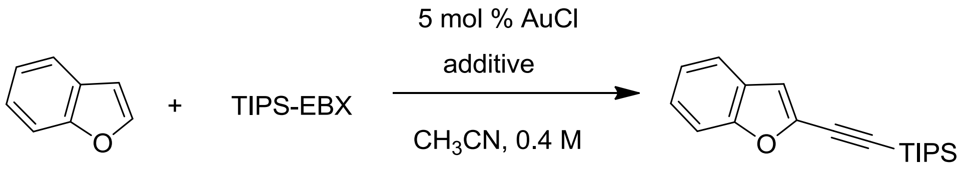

7a **8** **9a**

Equiv of TIPS-EBX (**8**)	Additive	Temp (°C)	Yield (%)
1.2	-	rt	0
1.2	-	60	0
1.2	1.2equiv $Zn(OTf)_2$	rt	22
1.2	1.2equiv $Zn(OTf)_2$	60	56
1.2	1.2equiv $Zn(OTf)_2$	40	48
1.2	1.2equiv $Zn(OTf)_2$	reflux	34
2	2.0equiv $Zn(OTf)_2$	60	75
2	2.0equiv $Zn(OTf)_2$	60 (0.1 M)	34
2	2.0equiv $Zn(OTf)_2$	60 (0.8 M)	68
2	2.0equiv TFA	60	34
2	2.0equiv $Zn(BF_4)_2$	60	0
2	0.2equiv $Zn(OTf)_2$	60	37
2	4.0 equiv $Zn(OTf)_2$	60	0
2	2.0equiv $Zn(NTf_2)_2$	60	57
2	2.0equiv $Yb(OTf)_3$	60	62
2	2.0equiv $Zn(CF_3COO)_2$	60	17
2	2.0equiv $ZnBr_2$	60	trace
2	2.0equiv $Zn(CH_3COO)_2$	60	9

General procedure (GP): TIPS-EBX (**8**, 342 mg, 0.800 mmol, 2.0 equiv), AuCl (4.6 mg, 0.020 mmol, 0.050 equiv), $Zn(OTf)_2$ (289 mg, 0.800 mmol, 2.0 equiv) and benzofuran derivatives **7** (0.40 mmol, 1.0 equiv) were added into CH_3CN (2.0 mL) under air. The mixture was stirred for 26 hours at 60 °C. Then the mixture was concentrated with silica gel and purified by column chromatography directly.

(Benzofuran-2-ylethynyl)triisopropylsilane (9a)

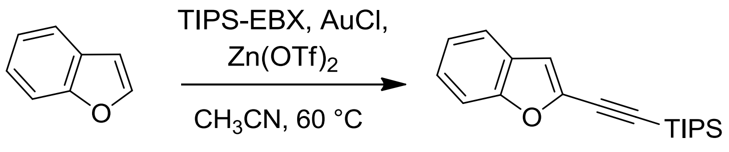

7a **9a**

Following the conditions described in **GP**, the crude product was purified by flash column chromatography (SiO_2; pentane) to afford **9a** as a yellow oil (90 mg, 0.30 mmol, 75%).

R_f (pentane): 0.6.
^{1}H NMR (400 MHz, $CDCl_3$) δ 7.54 (d, 1 H, *J* = 7.8 Hz, *ArH*), 7.45 (m, 1 H, *ArH*), 7.33 (m, 1 H, *ArH*), 7.21 (m, 1 H, *ArH*), 6.95 (s, 1 H, *FuranH*), 1.15 (m, 21 H, *TIPS*); ^{13}C NMR (101 MHz, $CDCl_3$) δ 154.6, 138.7, 127.5, 125.6, 123.2, 121.2, 111.9, 111.2, 98.7, 96.2, 18.6, 11.2; IR 2943 (s), 2865 (s), 2155 (w), 2154 (w), 1558 (w), 1490 (w), 1463 (m), 1449 (m), 1261 (w), 1181 (m), 1019 (w), 997 (m), 952 (w), 922 (w), 884 (s), 818 (w), 809 (w). HRMS (ESI) calcd for $C_{19}H_{26}OSi[M]^+$ 298.1753; found 298.1749.

Triisopropyl((5-methoxybenzofuran-2-yl)ethynyl)silane (9b)

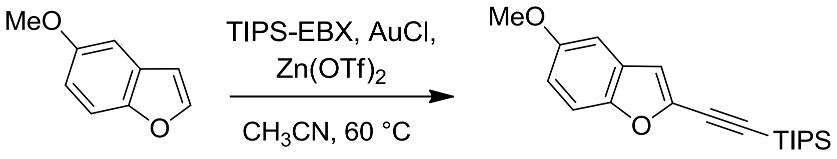

7b **9b**

Following the conditions described in **GP**, the crude product was purified by flash column chromatography (SiO_2; Pentane/DCM 100/1) to afford **9b** as a yellow oil (96 mg, 0.29 mmol, 73%).

R_f (pentane/DCM 100/1): 0.2.
^{1}H NMR (400 MHz, $CDCl_3$) δ 7.33 (d, 1 H, *J* = 8.8 Hz, *ArH*), 6.97 (d, 1 H, *J* = 2.5 Hz, *ArH*), 6.93 (dd, 1 H, *J* = 8.9, 2.6 Hz, *ArH*), 6.89 (s, 1 H, *FuranH*), 3.84 (s, 3 H, CH_3), 1.14 (m, 21 H, *TIPS*); ^{13}C NMR (151 MHz, $CDCl_3$) δ 156.2, 149.6, 139.4, 128.0, 114.7, 112.0, 111.7, 103.0, 98.5, 96.3, 55.8, 18.6, 11.2; IR 2946 (s), 2945 (s), 2866 (m), 2363 (w), 2156 (w), 1617 (w), 1470 (s), 1299 (w), 1254 (w), 1210 (s), 1184 (m), 1032 (w), 997 (w), 956 (w), 883 (w). HRMS (ESI) calcd for $C_{20}H_{29}O_2Si^+$ $[M+H]^+$ 329.1931; found 329.1917.

((5-Bromobenzofuran-2-yl)ethynyl)triisopropylsilane (9c)

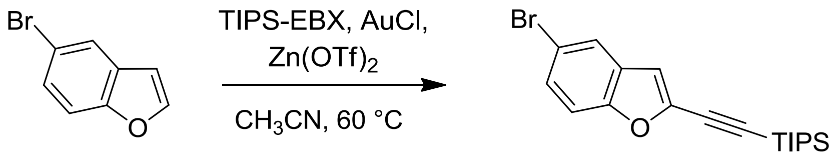

7c **9c**

Following the conditions described in **GP**, the crude product was purified by flash column chromatography (SiO_2; pentane) to afford **9c** as a yellow oil (96 mg, 0.26 mmol, 64%).

R_f (pentane): 0.7.
^{1}H NMR (400 MHz, $CDCl_3$) δ 7.67 (d, 1 H, *J* = 1.9 Hz, *ArH*), 7.41 (dd, 1 H, *J* = 8.7, 1.9 Hz, *ArH*), 7.31 (d, 1 H, *J* = 8.7 Hz, *ArH*), 6.88 (s, 1 H, *FuranH*), 1.15 (m, 21 H, *TIPS*); ^{13}C NMR (151 MHz, $CDCl_3$) δ 153.3, 139.9, 129.5, 128.5, 123.7, 116.3, 112.7, 111.1, 99.7, 95.6, 18.6, 11.2; IR 2945 (s), 2866 (s), 2362 (m), 2337 (w), 2159 (w), 1458 (s), 1321 (w), 1258 (w), 1206 (w), 1180 (s), 1070 (w), 1057 (w), 1015 (w), 997 (w), 952 (w), 881 (m). HRMS (ESI) calcd for $C_{38}Br_2H_{51}O_2Si_2^+$ $[M]^+$ 753.1789; found 753.1802.

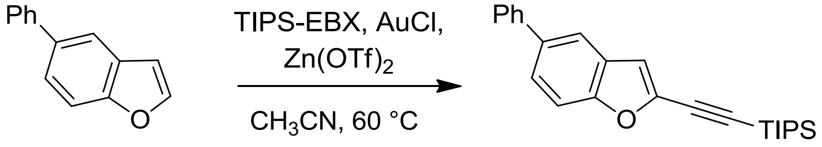

7d **9d**

Following the conditions described in **GP**, the crude product was purified by flash column chromatography (SiO_2; pentane/Et_2O 100/1) to afford **9d** as a colorless oil (106 mg, 0.290 mmol, 72%).

R_f (pentane/Et_2O 100/1): 0.7.
^{1}H NMR (400 MHz, $CDCl_3$) δ 7.72 (dd, 1 H, *J* = 1.8, 0.6 Hz, *ArH*), 7.60 (m, 2 H, *ArH*), 7.56 (dd, 1 H, *J* = 8.6, 1.9 Hz, *ArH*), 7.47 (m, 3 H, *ArH*), 7.34 (m, 1 H, *ArH*), 6.99 (d, 1 H, *J* = 0.9 Hz, *FuranH*), 1.17 (m, 21 H, *TIPS*); ^{13}C NMR (101 MHz, $CDCl_3$) δ 154.2, 141.3, 139.3, 136.9, 128.8, 128.1, 127.4, 127.0, 125.4, 119.5, 112.1, 111.3, 98.9, 96.1, 18.6, 11.2; IR 2945 (s), 2866 (s), 2363 (w), 2158 (w), 1462 (s), 1257 (w), 1240 (w), 1179 (m), 1073 (w), 1015 (w), 998 (w), 954 (w), 884 (m), 814 (w). HRMS (ESI) calcd for $C_{25}H_{30}OSi$ [M+] 374.2066; found . 374.2057.

((5-Hexylbenzofuran-2-yl)ethynyl)triisopropylsilane (9e)

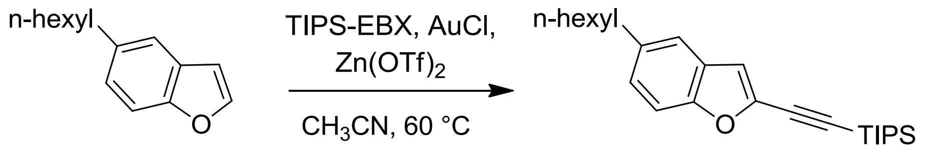

7e **9e**

Following the conditions described in **GP**, the crude product was purified by flash column chromatography (SiO_2; pentane) to afford **9e** as a colorless oil (77 mg, 0.20 mmol, 50%).

R_f (pentane): 0.6.
^{1}H NMR (400 MHz, $CDCl_3$) δ 7.34 (d, 1 H, *J* = 8.4, *ArH*), 7.32 (d, 1 H, *J* = 0.9 Hz, *ArH*), 7.14 (dd, 1 H, *J* = 8.4, 1.7 Hz, *ArH*), 6.89 (d, 1 H, *J* = 0.9 Hz, *FuranH*), 2.67 (t, 2 H, *J* = 7.5 Hz, *HexylH*), 1.63 (m, 2 H, *HexylH*), 1.36-1.26 (m, 6 H, *HexylH*), 1.14 (m, 21 H, *TIPS*), 0.88 (t, 3 H, *J* = 6.8 Hz, *HexylH*); ^{13}C NMR (101 MHz, $CDCl_3$) δ 153.2, 138.7, 137.9, 127.5, 126.4, 120.2, 111.8, 110.7, 98.3, 96.4, 35.8, 32.0, 31.7, 28.9, 22.6, 18.6, 14.1, 11.2. IR 2946 (m),), 2868 (m), 2361 (s), 2157 (w), 2114 (w), 1758 (w), 1727 (w), 1467 (w), 1264 (w), 1233 (w), 1195 (w), 995 (w), 884 (w), 812 (w), 729 (m). HRMS (ESI) calcd for $C_{25}H_{39}OSi^+$ $[M+H]^+$ 383.2765; found 383.2782.

((7-Bromobenzofuran-2-yl)ethynyl)triisopropylsilane (9f)

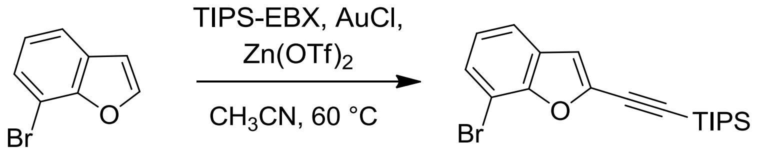

7f **9f**

Following the conditions described in **GP**, the crude product was purified by flash column chromatography (SiO_2; Pentane) to afford **9f** as a colorless oil (119 mg, 0.316 mmol, 79%).

R_f (pentane): 0.4.
^{1}H NMR (400 MHz, $CDCl_3$) δ 7.49 (dd, *J* = 4.9, 1.0 Hz, *ArH*), 7.47 (dd, 1 H, *J* = 4.9, 1.0 Hz, *ArH*), 7.11 (t, 1 H, *J* = 7.8 Hz, *ArH*), 7.00 (s, 1 H, *FuranH*), 1.19 (m, 21 H, *TIPS).* ^{13}C NMR (101 MHz, $CDCl_3$) δ 151.7, 139.5, 128.8, 128.5, 124.5, 120.2, 112.6, 103.9, 99.9, 95.5, 18.6, 11.2. IR 2945 (m), 2866 (m), 2361 (w), 2158 (w), 1579 (w), 1465 (w), 1417 (w), 1304 (w), 1223 (w), 1176 (m), 1137 (w), 1018 (w), 927 (m), 912 (w), 884 (w), 815 (w), 769 (w), 737 (s). HRMS (ESI) calcd for $C_{38}Br_2H_{51}O_2Si_2^+$ $[2M+H]^+$ 753.1789; found 753.1825.

Triisopropyl((7-phenylbenzofuran-2-yl)ethynyl)silane (9g)

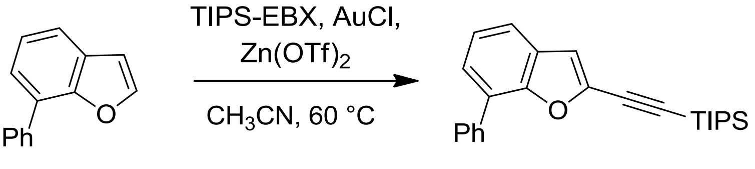

7g **9g**

Following the conditions described in **GP**, the crude product was purified by flash column chromatography (SiO_2; Pentane) to afford **9g** as a colorless oil (98 mg, 0.26 mmol, 65%).

R_f (pentane): 0.3.
^{1}H NMR (400 MHz, $CDCl_3$) δ 7.87 (m, 2 H, *ArH*), 7.50 (m, 4 H, *ArH*), 7.40 (m, 1 H, *ArH*), 7.32 (t, 1 H, *J* = 7.6 Hz, *ArH*), 7.02 (s, 1 H, *FuranH*), 1.16 (m, 21 H, *TIPS*). ^{13}C NMR (101 MHz, $CDCl_3$) δ 151.8, 138.9, 136.2, 128.7, 128.6, 128.4, 127.7, 125.4, 125.1, 123.8, 120.2, 112.3, 98.8, 96.3, 18.6, 11.2. IR 2945 (s), 2892 (w), 2866 (s), 2361 (s), 2156 (w), 1730 (w), 1685 (w), 1564 (w), 1465 (m), 1409 (w), 1249 (w), 1210 (m), 1171 (w), 1017 (w), 998 (w), 956 (w), 912 (w), 884 (m), 820 (w), 757 (s). HRMS (ESI) calcd for $C_{25}H_{31}OSi^+$ $[M+H]^+$ 375.2139; found 375.2142.

Triisopropyl((3-methylbenzofuran-2-yl)ethynyl)silane (9h)

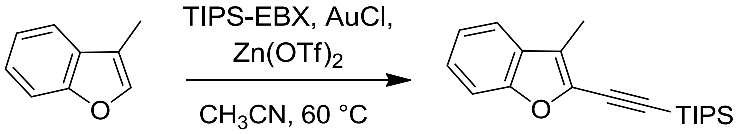

7h **9h**

Following a slightly modified version of **GP**, TIPS-EBX (**8**) (514 mg, 1.20 mmol, 3.0 equiv) and $Zn(OTf)_2$ (434 mg, 1.20 mmol, 3.0 equiv) were used. The crude product was purified by flash column chromatography (SiO_2; pentane) to afford **9h** as a colorless oil (72 mg, 0.23 mmol, 58%).

R_f (pentane): 0.5.
^{1}H NMR (400 MHz, $CDCl_3$) δ 7.52 (d, 1 H, *J* = 7.7 Hz *ArH*), 7.45 (d, 1 H, *J* = 8.0 Hz *ArH*), 7.36 (t, 1 H, *J* = 8.0 Hz *ArH*), 7.29 (d, 1 H, *J* = 9.0 Hz *ArH*), 2.37 (s, 3 H, CH_3), 1.19 (m, 21 H, *TIPS*); ^{13}C NMR (101 MHz, $CDCl_3$) δ 154.1, 136.0, 128.7, 125.6, 122.7, 121.9, 119.6, 111.2, 100.8, 95.8, 18.6, 11.2, 8.9; IR 2943 (m), 2865 (m), 2154 (w), 1745 (w), 1463 (m), 1449 (s), 1387 (w), 1353 (w), 1281 (w), 1250 (w), 1191 (s), 1116 (w), 1096 (m), 1071 (w), 1017 (w), 997 (w), 920 (w), 883 (s). HRMS (ESI) calcd for $C_{20}H_{29}OSi^+$ $[M+H]^+$ 313.1982; found 313.1981.

Triisopropyl((7-methoxybenzofuran-4-yl)ethynyl)silane (9j) + triisopropyl((7-methoxy-benzofuran-6-yl)ethynyl)silane (9j')

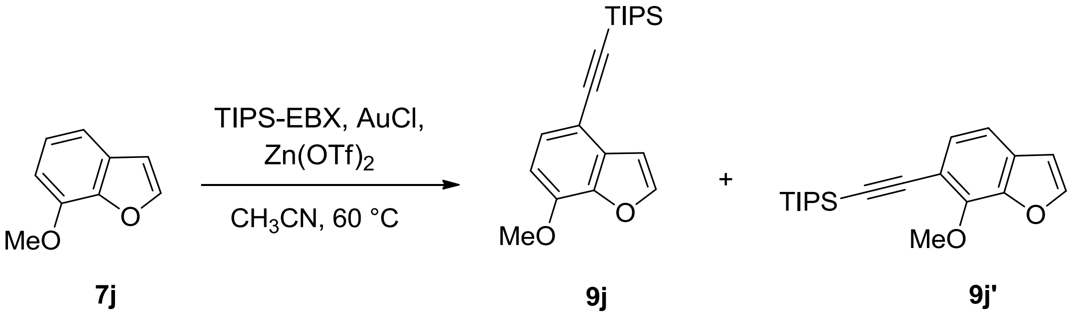

Following the conditions described in **GP**, the crude product was purified by flash column chromatography (SiO_2; pentane/DCM 100/1) to afford an inseparable mixture of two isomers **9j** and **9j'** as yellow oil (66 mg, 0.20 mmol, 50%).

R_f (pentane/DCM 100/1): 0.5.
^{1}H NMR (400 MHz, $CDCl_3$) δ 7.60 (d, 0.2 H, J = 2.1 Hz, *ArH* minor isomer), 7.58 (d, 1 H, J = 2.1 Hz, *ArH* major isomer), 7.32 (d, 0.2 H, J = 8.3 Hz, *FuranH*, minor isomer), 7.25 (d, 1 H, J = 8.3 Hz, *FuranH*, major isomer), 6.90 (d, 0.2 H, J = 2.1 Hz, *ArH* minor isomer), 6.82 (d, 1 H, J = 2.1 Hz, *ArH* major isomer), 6.67 (d, 0.2H, J = 8.3 Hz, *FuranH*, minor isomer), 6.66 (d, 1H, J = 8.3 Hz, *FuranH*, major isomer) 4.03 (s, 0.6 H, *OCH_3*, minor isomer), 4.02 (s, 3 H, *OCH_3*, major isomer), 1.16 (s, 21 H, *TIPS*, major isomer), 1.13 (s, 4 H, *TIPS*, minor isomer). ^{13}C NMR (151 MHz, $CDCl_3$) δ 146.8, 145.9, 145.8, 145.4, 143.4, 131.9, 131.4, 129.6, 127.9, 108.5, 106.9, 106.7, 106.5, 106.2, 104.5, 91.9, 89.7, 88.0, 76.1, 73.6, 56.2, 56.1, 29.7, 18.7, 18.6, 11.3. IR 2943 (s), 2865 (s), 2362 (m), 2147 (m), 1619 (m), 1584 (w), 1497 (s), 1464 (m), 1407 (w), 1336 (w), 1289 (s), 1258 (w), 1199 (w), 1176 (w), 1093 (m), 1044 (m), 915 (w), 882 (m). HRMS (ESI) calcd for $C_{20}H_{29}O_2Si^+$ $[M+H]^+$ 329.1931; found 329.1928.

2-((Triisopropylsilyl)ethynyl)-9-methoxy-7*H*-furo[3,2-*g*]chromen-7-one (10)

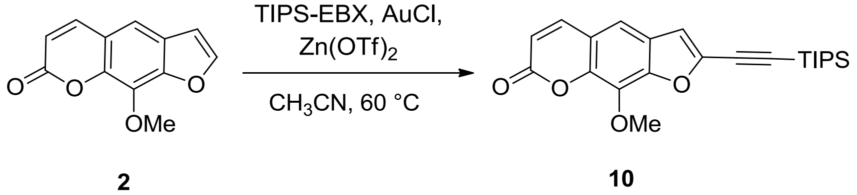

Following the conditions described in **GP**, the crude product was purified by flash column chromatography (SiO_2; pentane/EA 10/1) to afford **10** as yellow oil (58 mg, 0.15 mmol, 37%).

R_f (pentane/EA 10/1): 0.7.

^{1}H NMR (400 MHz, $CDCl_3$) δ 8.22 (d, 1 H, J = 9.7 Hz, *VinylH*), 7.71 (s, 1 H, *FuranH*), 6.94 (s, 1 H, *ArH*), 6.45 (d, 1 H, J = 9.7 Hz, *VinylH*), 4.33 (s, 3 H, *CH3*), 1.18 (m, 21 H, *TIPS*). ^{13}C NMR (101 MHz, $CDCl_3$) δ 160.1, 146.9, 146.4, 142.9, 142.3, 133.4, 129.3, 117.8, 115.4, 106.9, 106.7, 100.4, 100.1, 61.4, 18.7, 11.2. IR 2944 (w), 2866 (w), 2146 (w), 1742 (s), 1587 (m), 1473 (m), 1423 (w), 1380 (w), 1310 (w), 1134 (s), 1046 (s), 1028 (w), 916 (w), 830 (w), 814 (w). HRMS (ESI) calcd for $C_{23}H_{28}NaO_4Si^+$ $[M+Na]^+$ 419.1649; found 419.1657.

2-Ethynyl-9-methoxy-7*H*-furo[3,2-*g*]chromen-7-one (13)

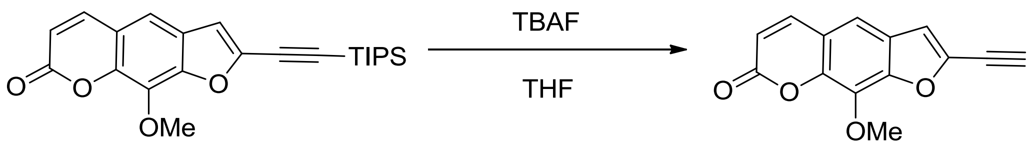

10

13

290 µL TBAF (1M in THF, 0.29 mmol, 2.0 equiv) was added into a solution of **10** (53.0 mg, 0.145 mmol, 1.0 equiv) in 2 mL dry THF. The solution was kept overnight then was quenched with sat NH_4Cl (10 mL). The aqueous phase was extracted with DCM (3 × 5 mL). The combined organic phase was dried over $MgSO_4$ and concentrated. The crude product was purified by flash column chromatography (SiO_2; Pentane/EA 5/1) to afford **13** as yellow solid. The yellow solid was washed by pentane to get pure compround (8 mg, 0.04 mmol, 26%).

R_f: (pentane/EA 10/1): 0.4

Melting point: 248–249 °C.

^{1}H NMR (400 MHz, $CDCl_3$) δ 7.75 (d, 1 H, J = 9.6 Hz, *VinylH*), 7.32 (s, 1 H, *ArH*), 7.03 (s, 1 H, *FuranH*), 6.39 (d, 1 H, J = 9.6 Hz, *VinylH*), 4.30 (s, 3 H, *CH3*), 3.56 (s, 1 H, *CH*). ^{13}C NMR (101 MHz, $CDCl_3$) δ 160.1, 147.4, 144.1, 144.0, 139.6, 132.5, 125.7, 117.0, 115.2, 113.0, 112.3, 84.7, 73.2, 61.4. IR 3203 (w), 2363 (w), 1713 (s), 1620 (w), 1468 (w), 1400 (w), 1324 (w), 1267 (w), 1237 (w), 1219 (w), 1164 (w), 1021 (w), 962 (w), 936 (w), 874 (w), 815 (m), 776 (w), 760 (w). HRMS (ESI) calcd for $C_{14}H_9O_4^+$ $[M+H]^+$ 241.0495; found 241.0491.

NMR Experiment

TIPS-EBX (**8**, 43 mg, 0.10 mmol) and $Zn(OTf)_2$ (36 mg, 0.10 mmol) were mixed in $CDCl_3$ (1 mL).

^{1}H NMR (400 MHz, $CDCl_3$) δ 8.58 (m, 1 H, *ArH*), 8.20 (m, 1 H, *ArH*), 7.76 (m, 2 H, *ArH*), 1.18 (m, 21 H, *TIPS*).

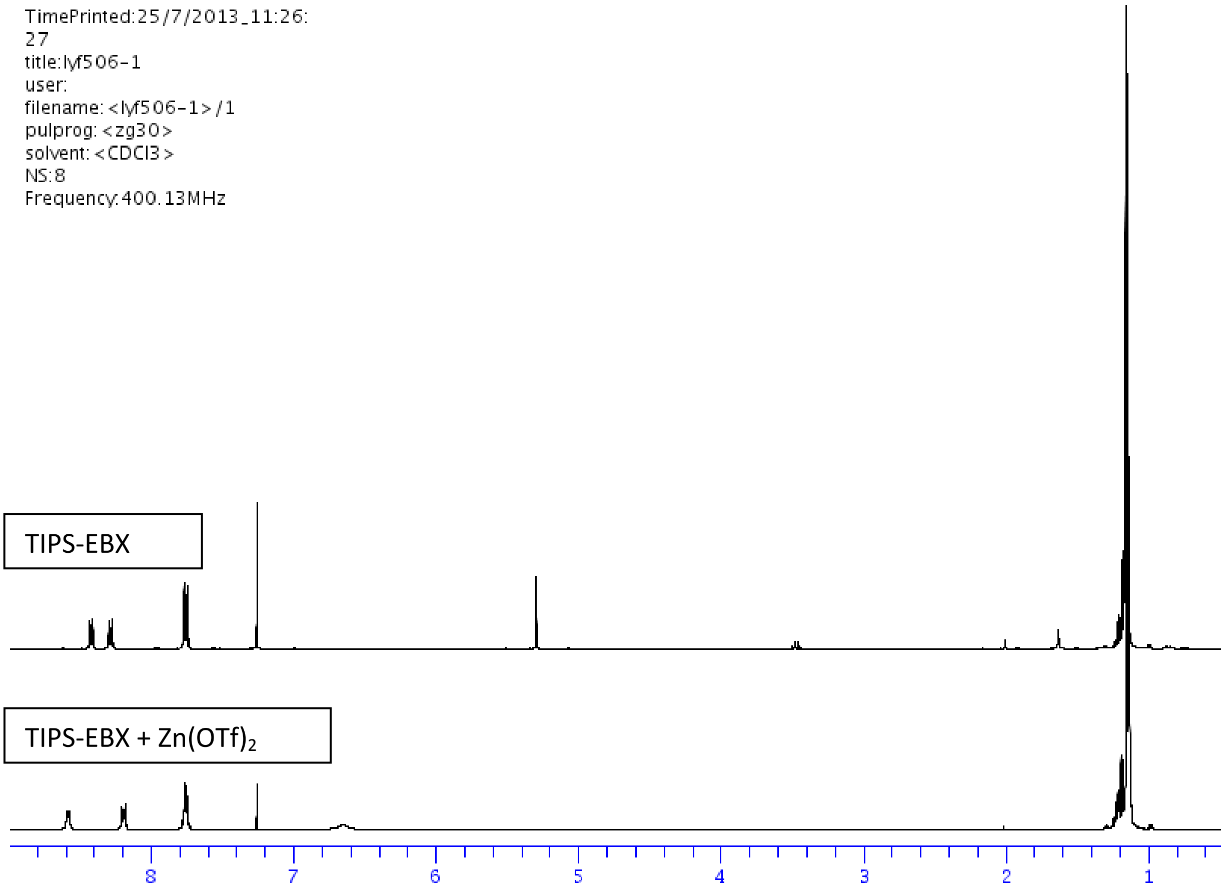

Figure S1: ^{1}H NMR spectrum of Zn(OTF)$_2$/TIPS-EBX (**8**) adduct

5. Spectra of New Compounds

solvent: <CDCl3>
Frequency: 400.13MHz

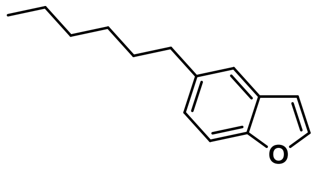

7e

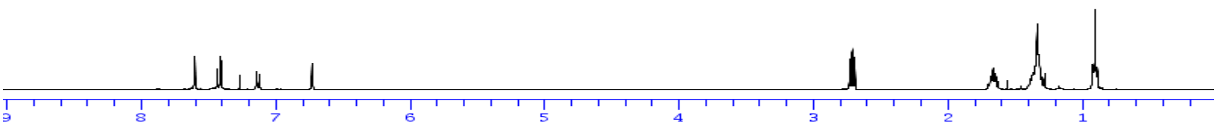

solvent: <CDCl3>
Frequency: 100.612769MHz

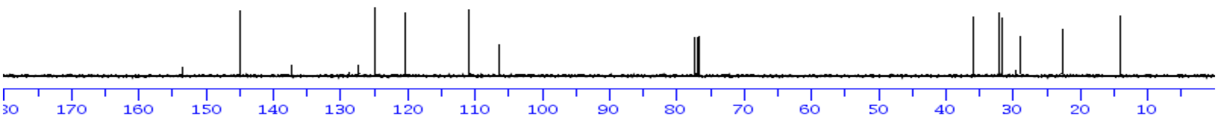

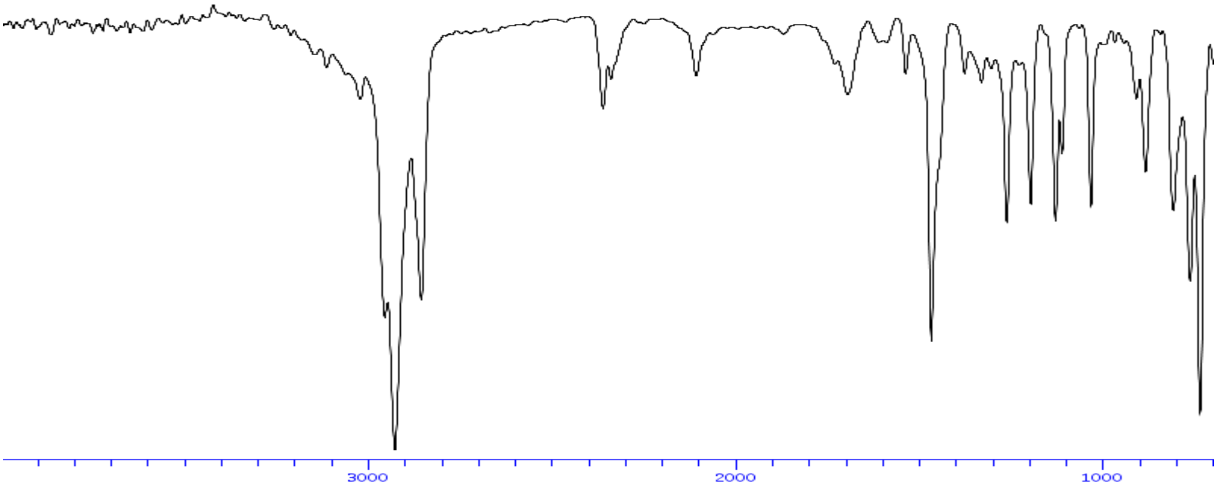

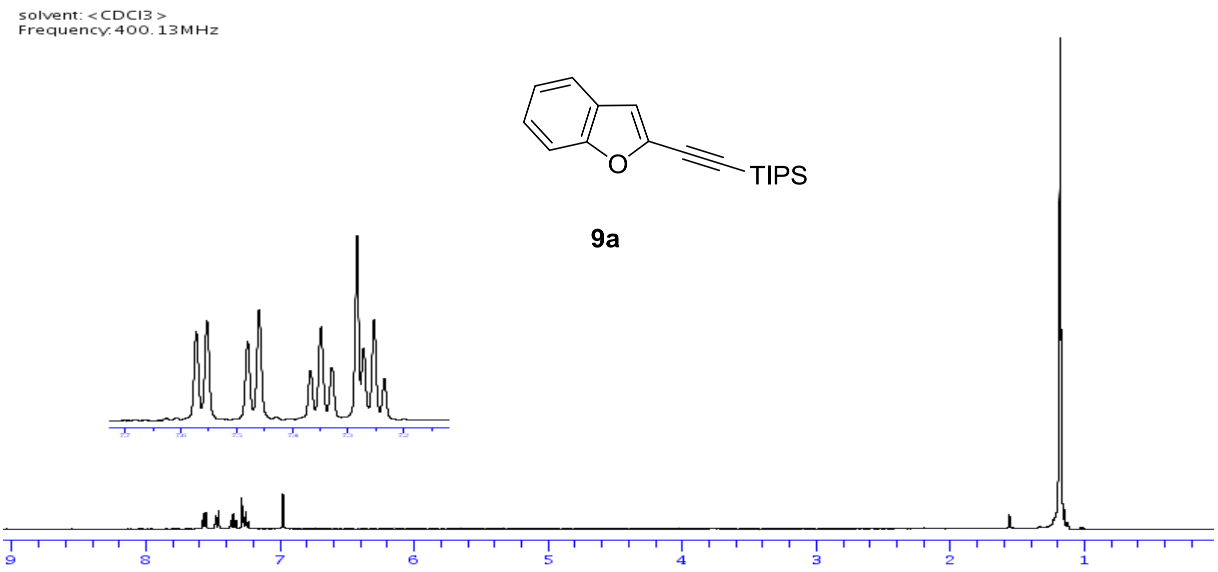
solvent: <CDCl3>
Frequency: 400.13MHz
TIPS
O
9a

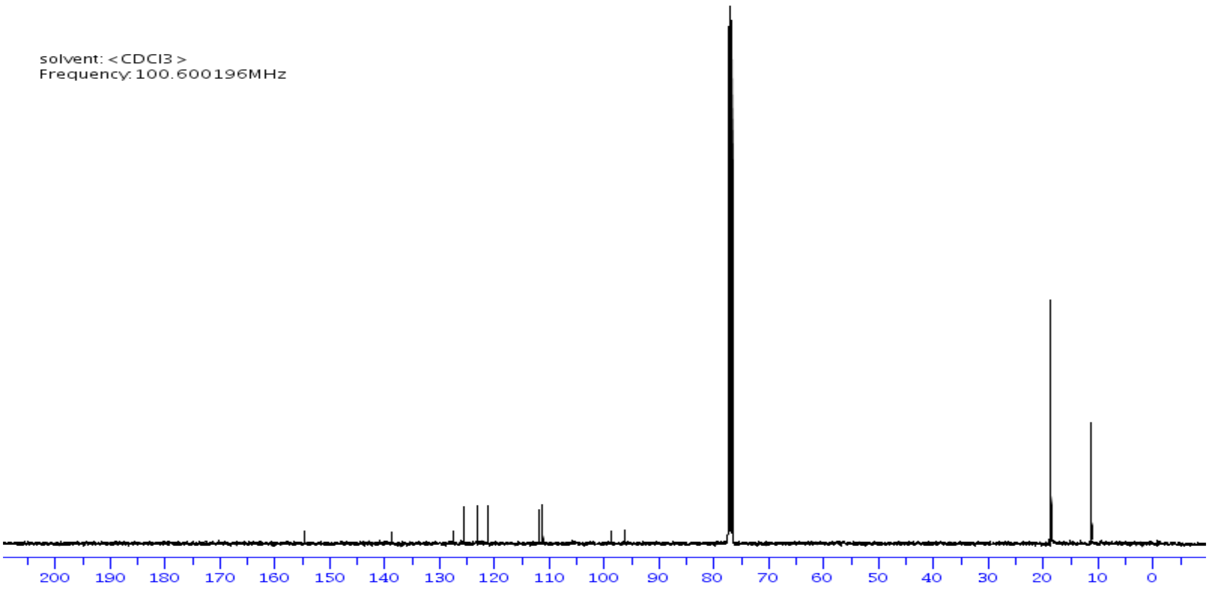
solvent: <CDCl3>
Frequency: 100.600196MHz

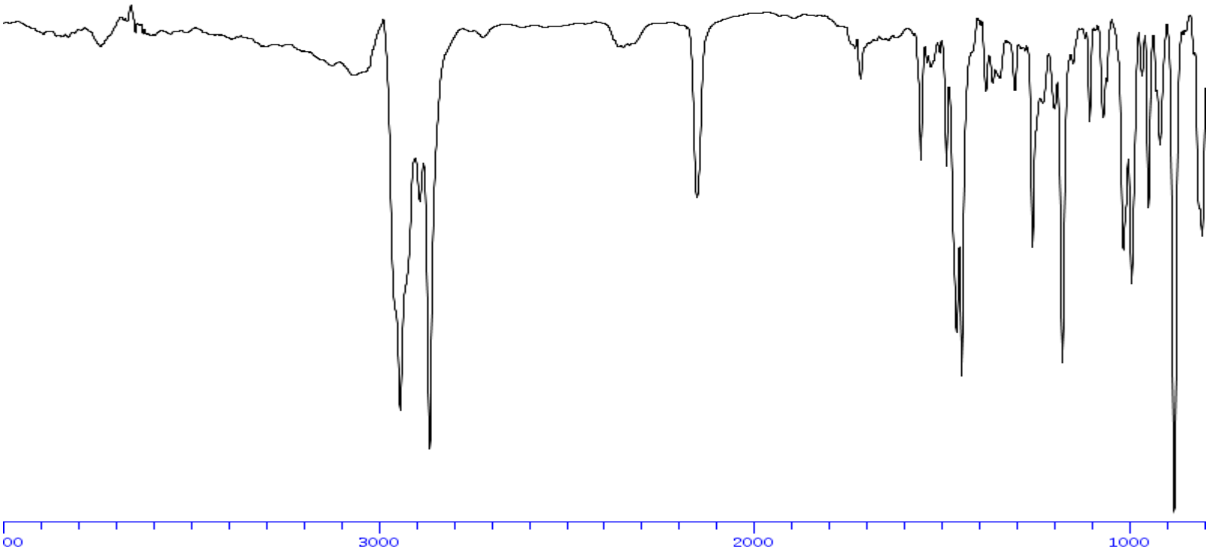

solvent: <CDCl3>
Frequency: 400.13MHz

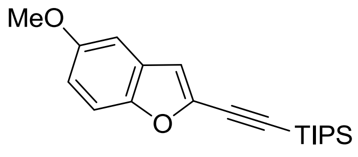

9b

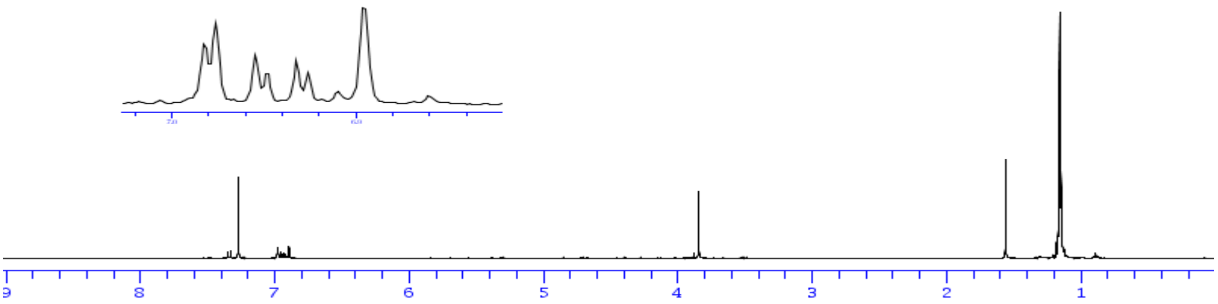

solvent: <CDCl3>
Frequency: 150.902809MHz

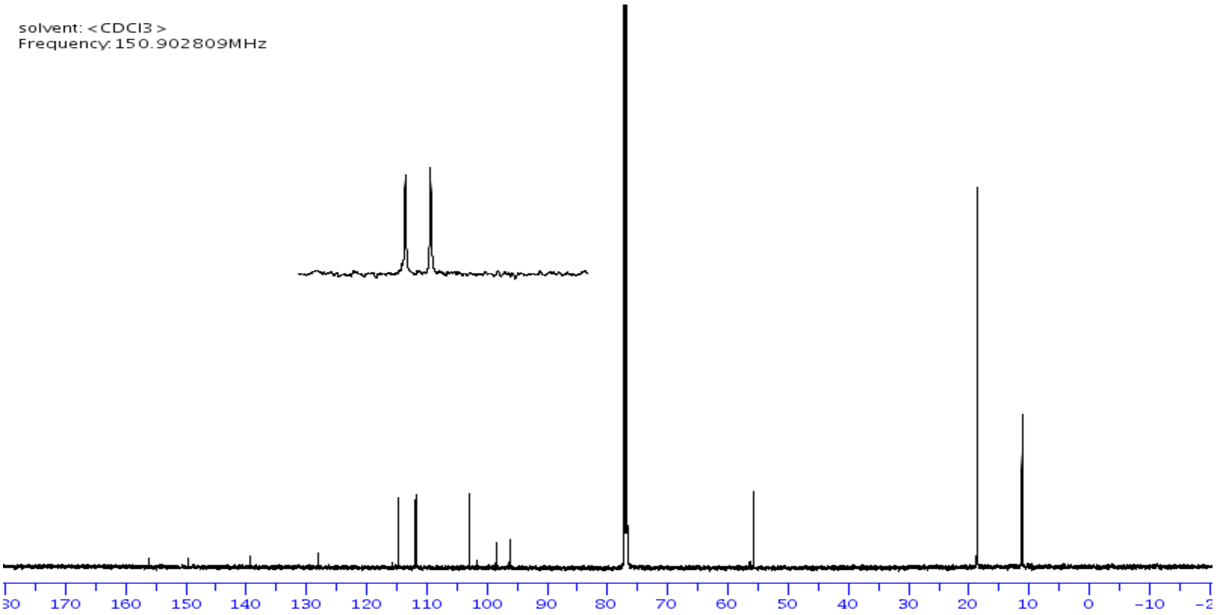

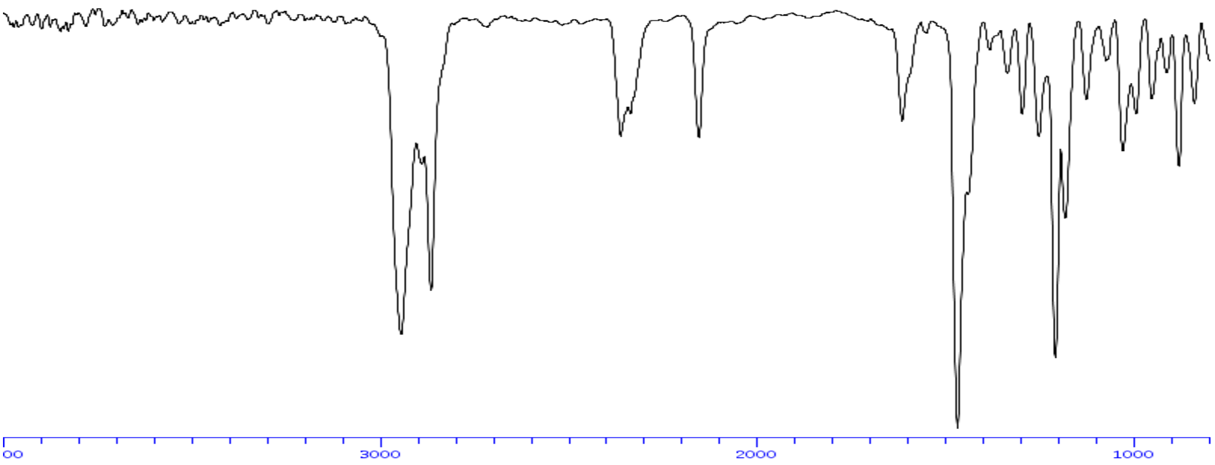

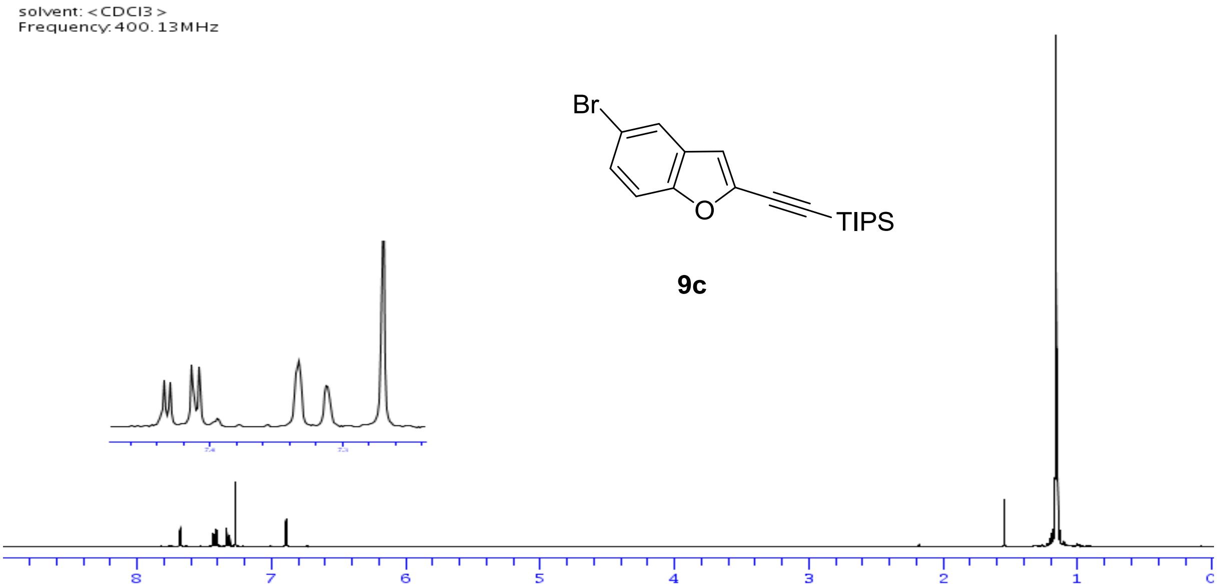
solvent: <CDCl3>
Frequency: 400.13MHz
Br
O
TIPS

9c

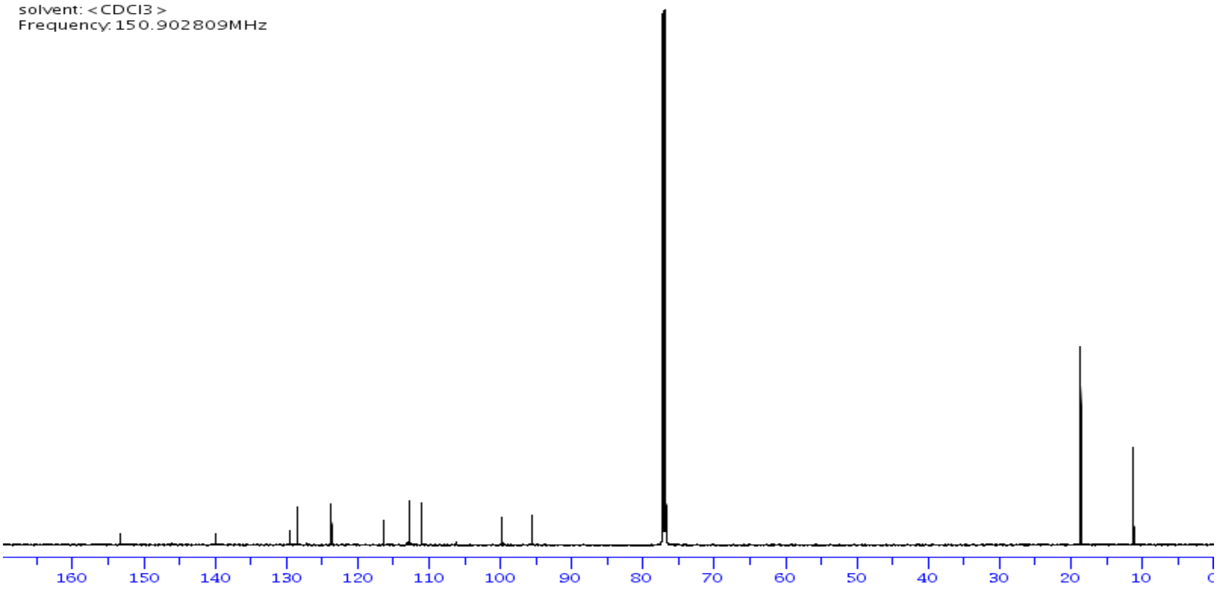
solvent: <CDCl3>
Frequency: 150.902809MHz

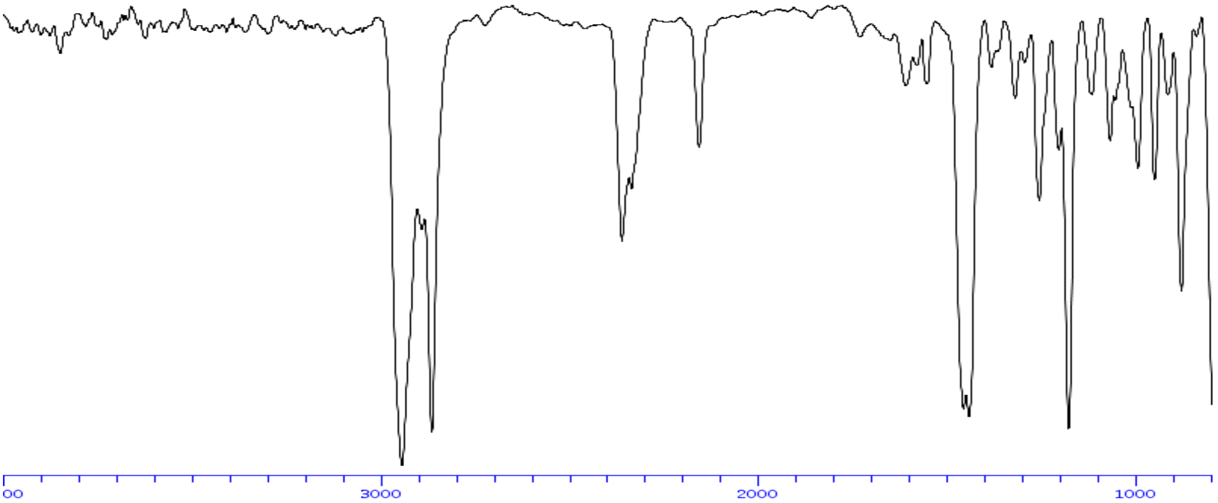

solvent: <CDCl3>
Frequency: 400.13MHz

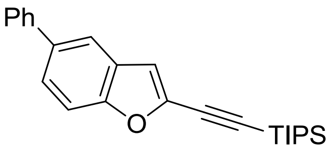

9d

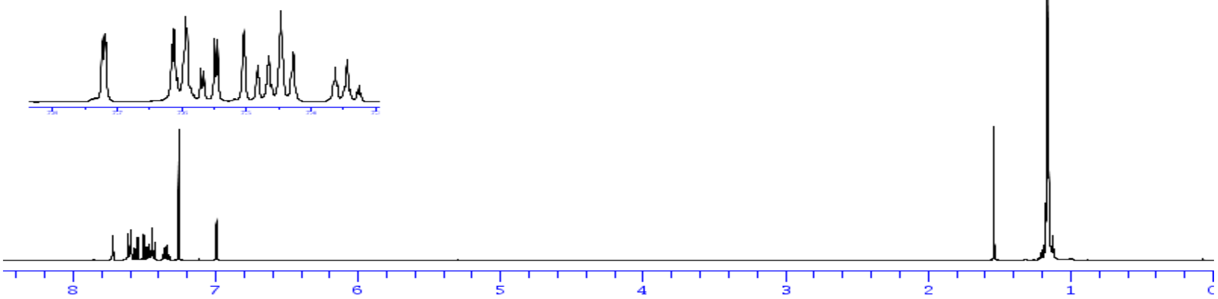

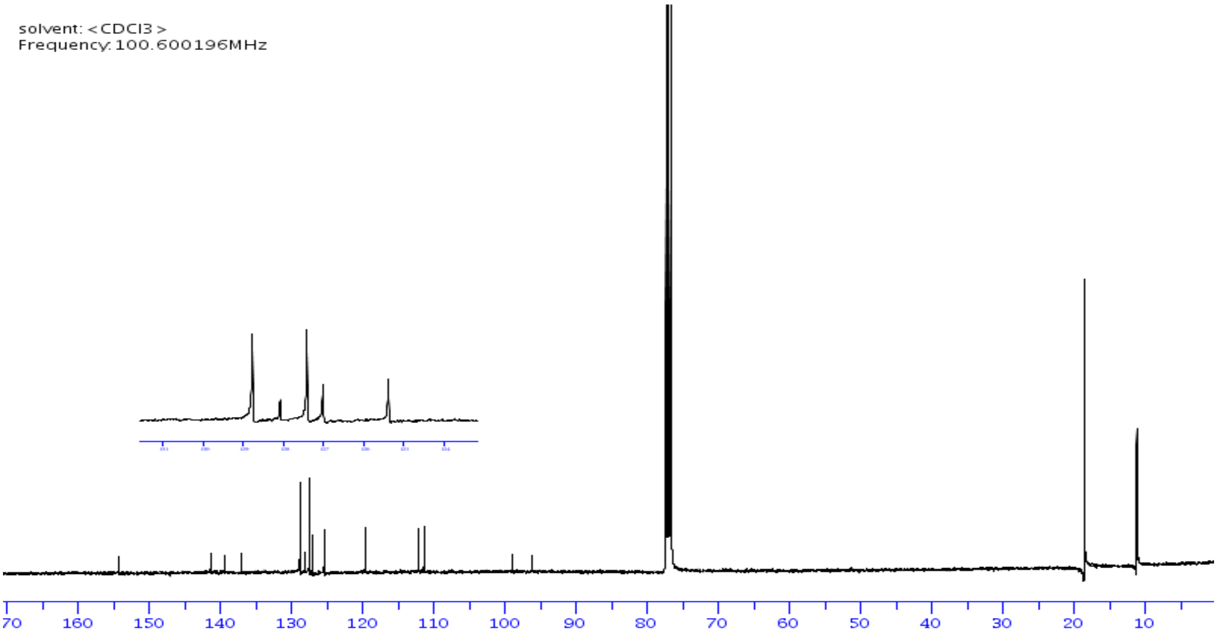

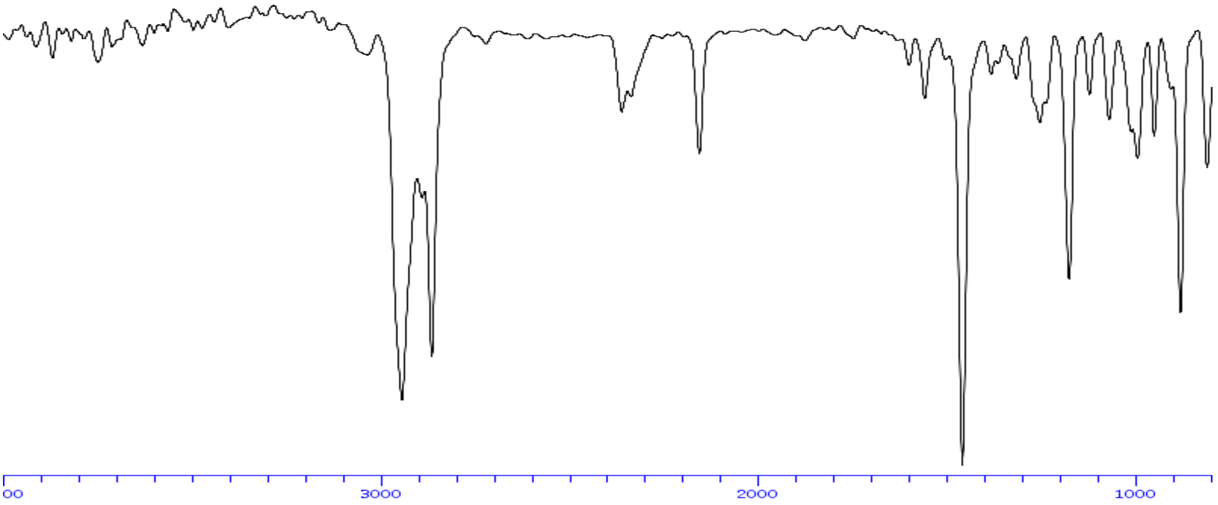

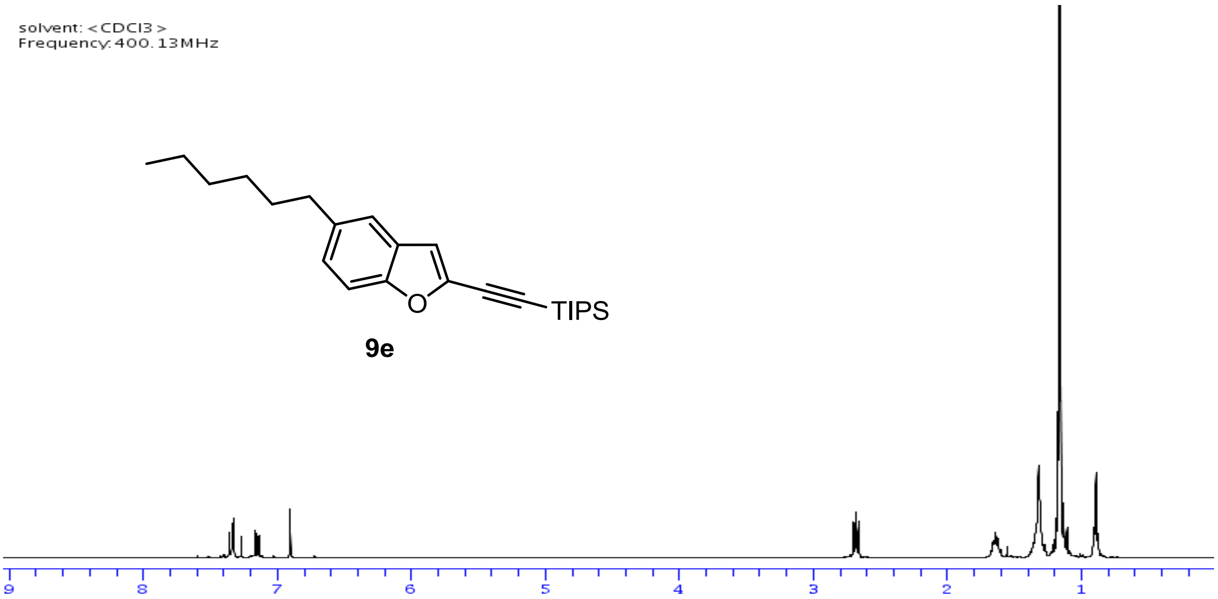
solvent: <CDCl3>
Frequency: 400.13MHz
TIPS
9e
9 8 7 6 5 4 3 2 1

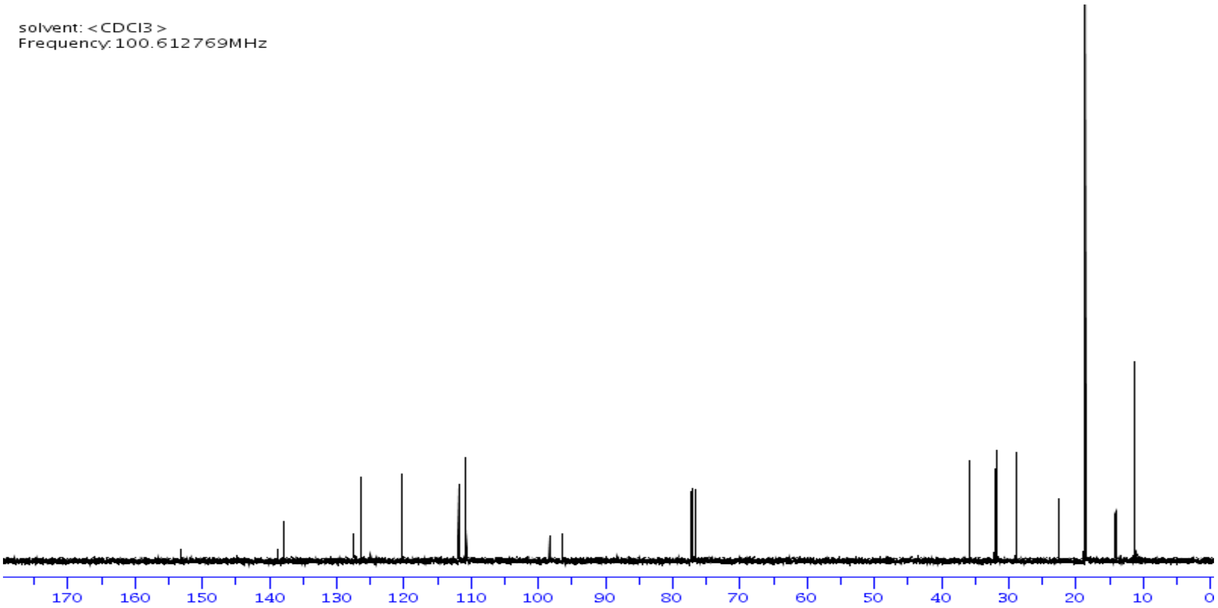
solvent: <CDCl3>
Frequency: 100.612769MHz
170 160 150 140 130 120 110 100 90 80 70 60 50 40 30 20 10 0

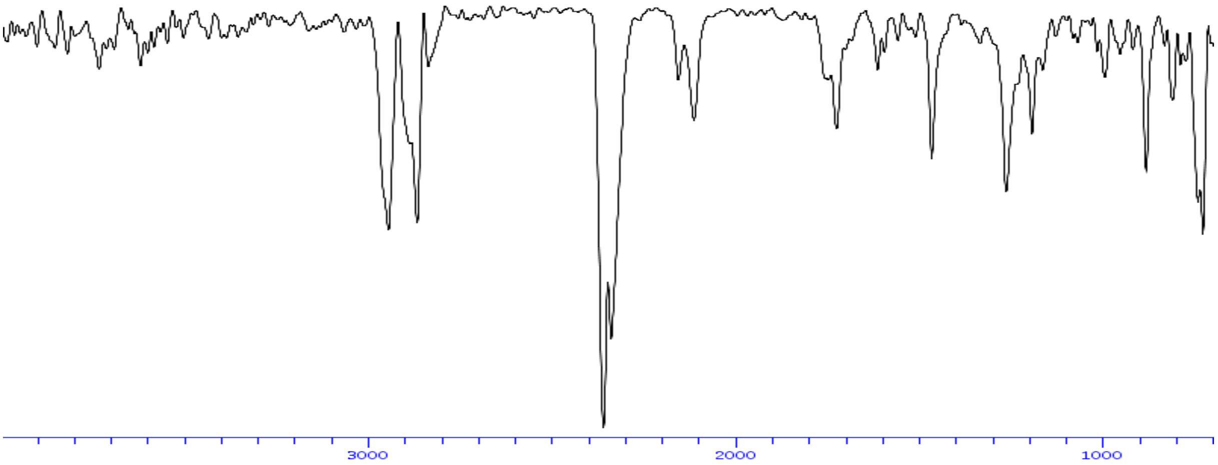
3000 2000 1000

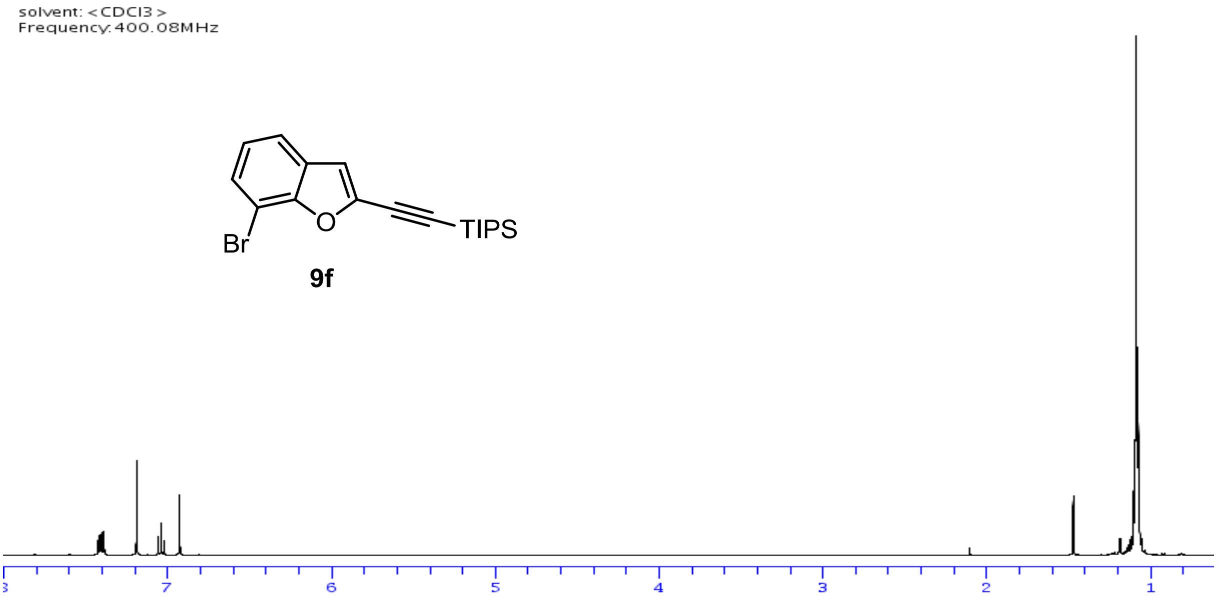
solvent: <CDCl3>
Frequency: 400.08MHz
Br
O
TIPS
9f
7
6
5
4
3
2
1

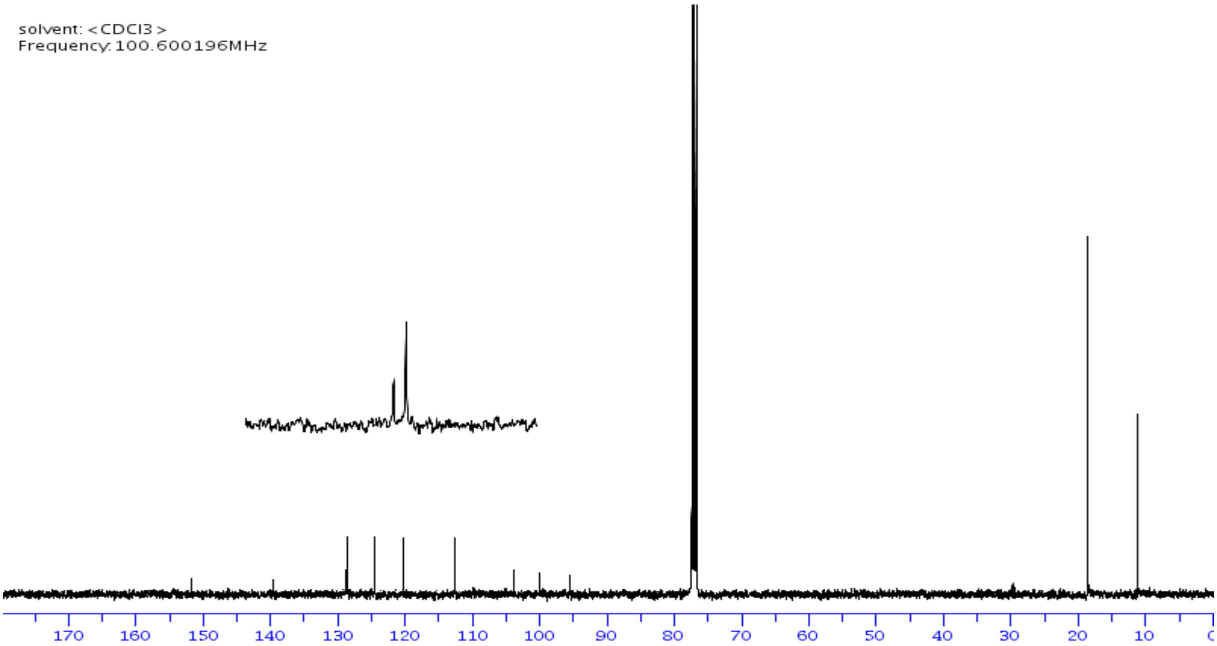
solvent: <CDCl3>
Frequency: 100.600196MHz
170 160 150 140 130 120 110 100 90 80 70 60 50 40 30 20 10

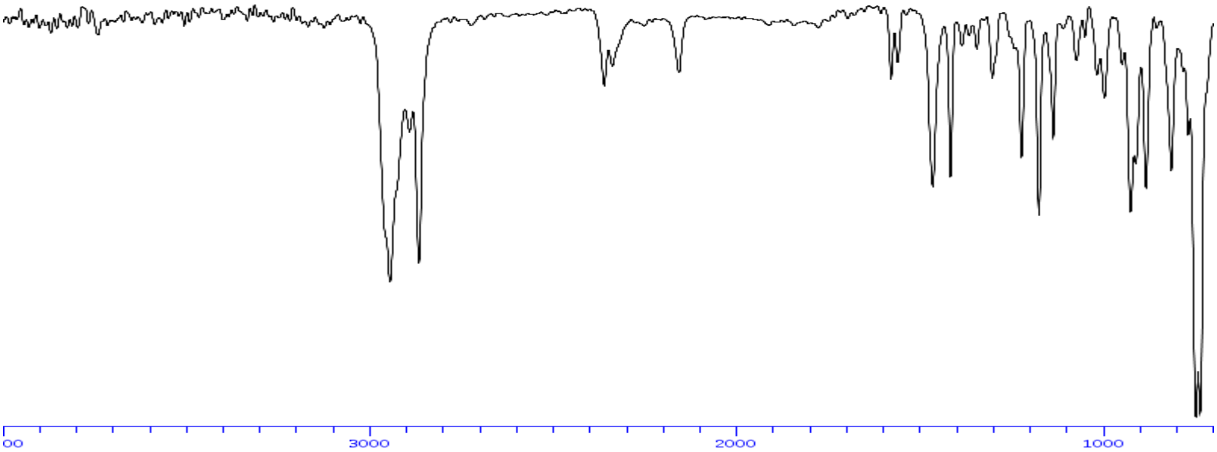
3000
2000
1000

solvent: <CDCl3>
Frequency: 400.13MHz

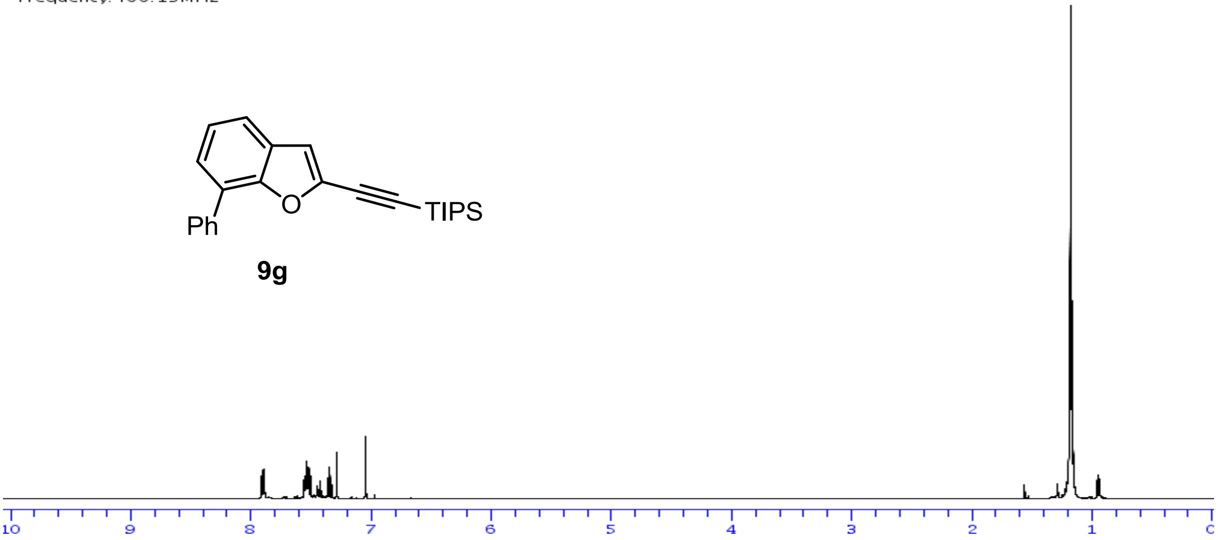

9g

solvent: <CDCl3>
Frequency: 100.600196MHz

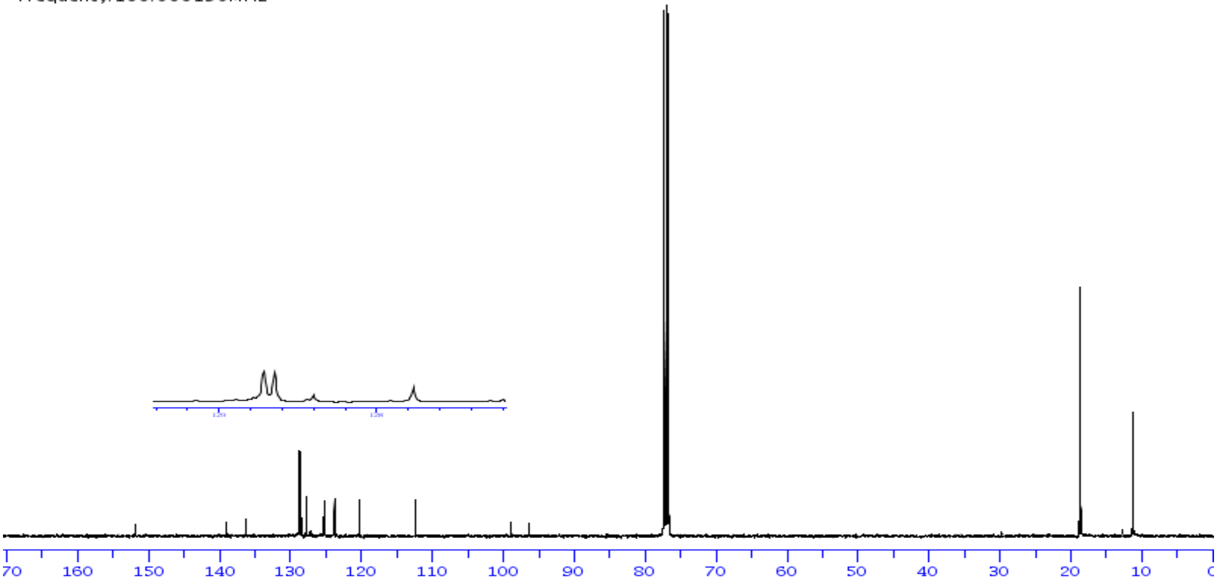

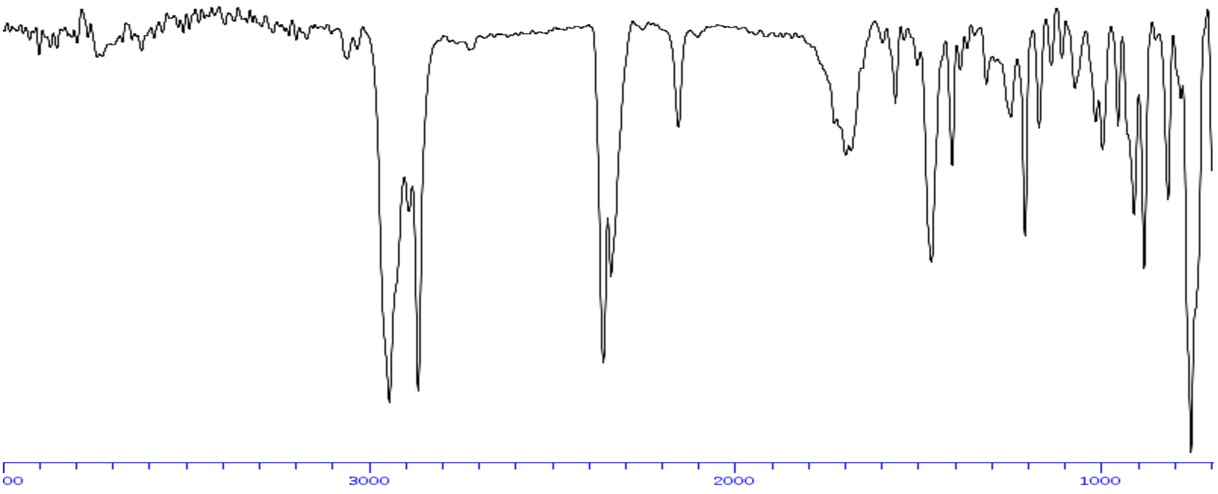

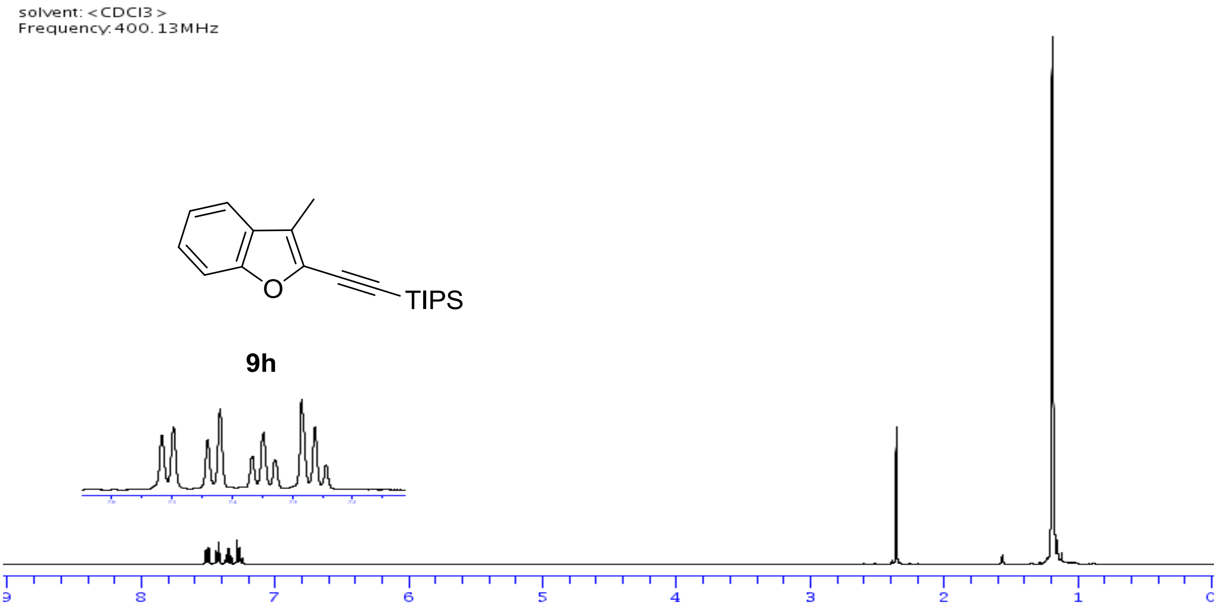
solvent: <CDCl3>
Frequency: 400.13MHz
TIPS
O
9h
9
8
7
6
5
4
3
2
1
0

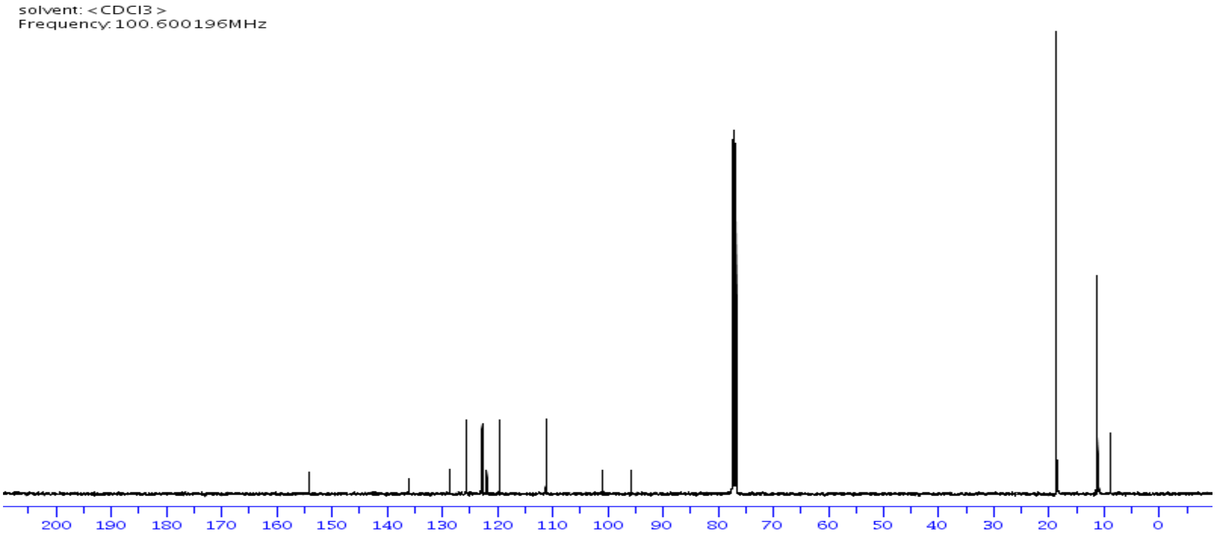
solvent: <CDCl3>
Frequency: 100.600196MHz
200
190
180
170
160
150
140
130
120
110
100
90
80
70
60
50
40
30
20
10
0

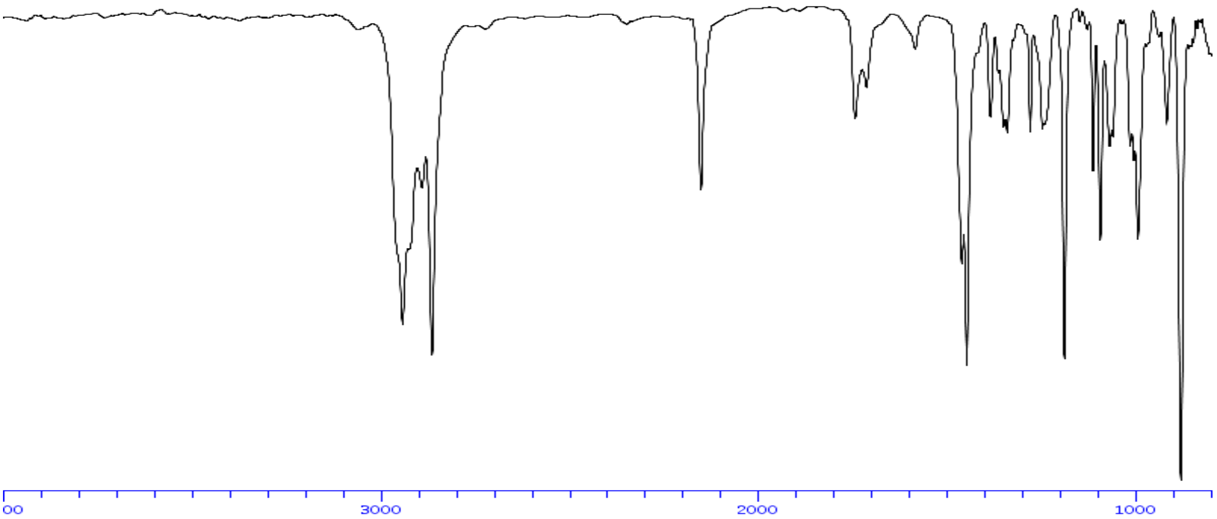
3000
2000
1000

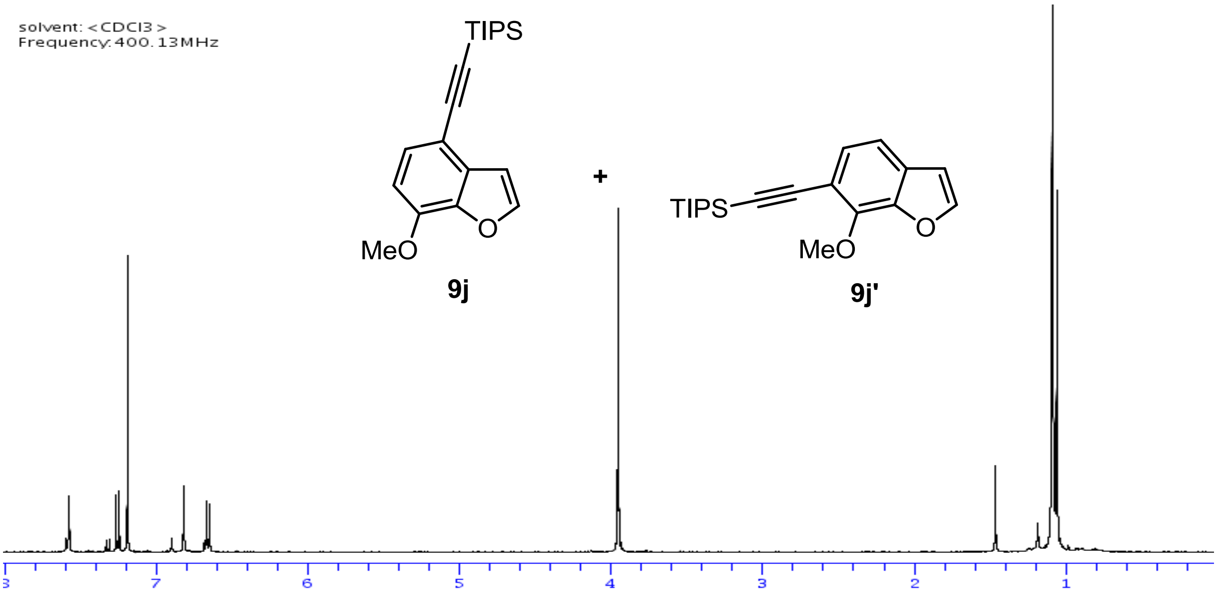
solvent: <CDCl3>
Frequency: 400.13MHz
TIPS
MeO
O
9j
+
TIPS
MeO
O
9j'
7
6
5
4
3
2
1

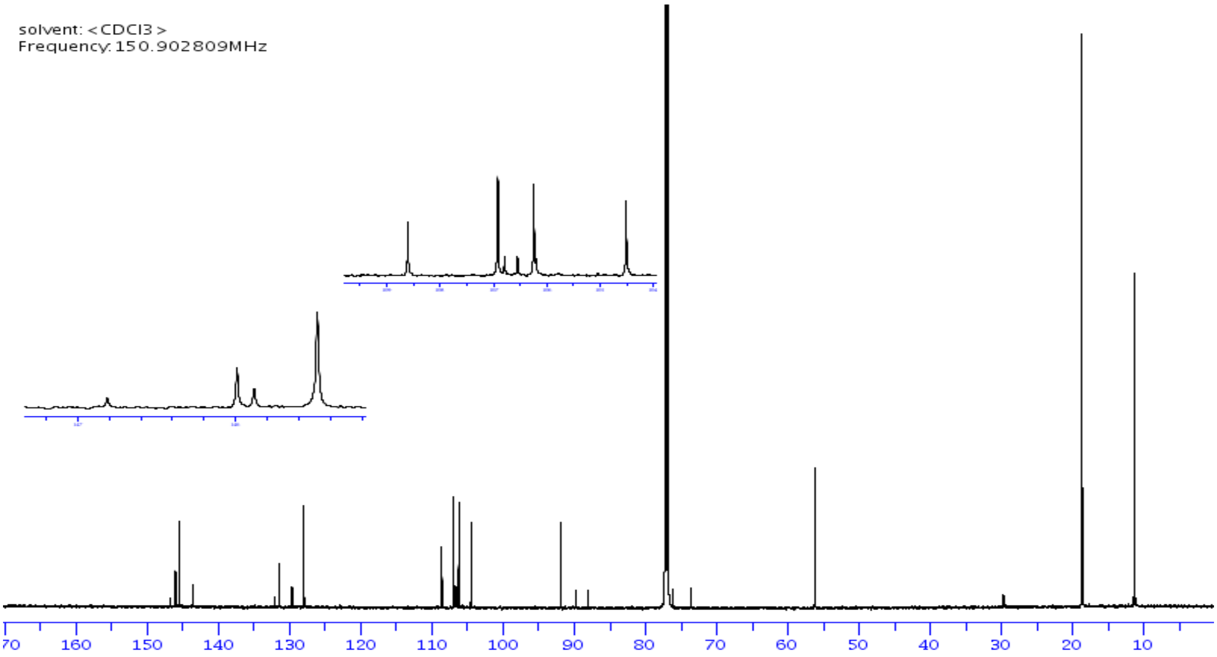
solvent: <CDCl3>
Frequency: 150.902809MHz
160
150
140
130
120
110
100
90
80
70
60
50
40
30
20
10

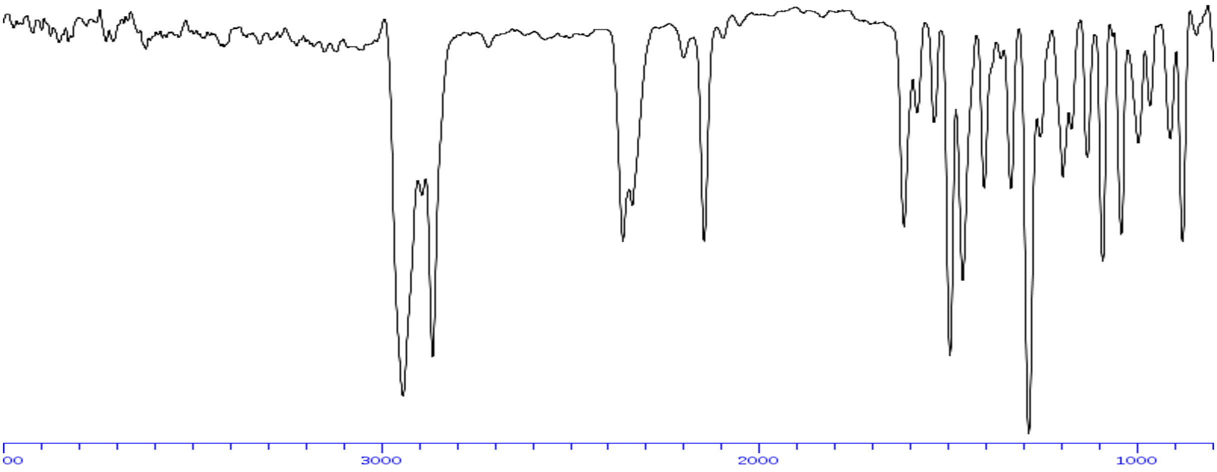
3000
2000
1000

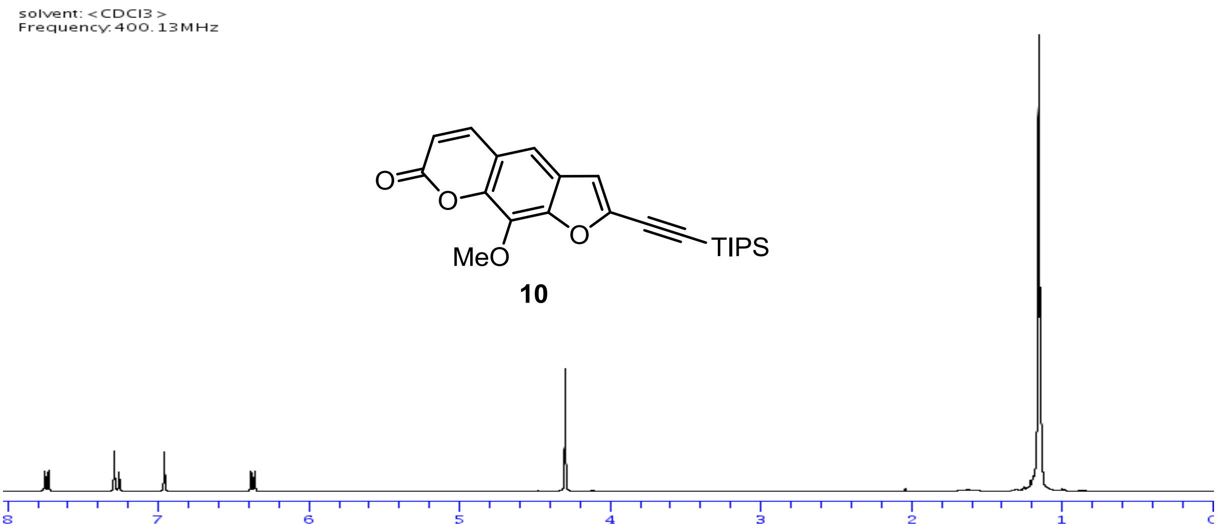
solvent: <CDCl3>
Frequency: 400.13MHz
O
O
MeO
O
TIPS
10
8
7
6
5
4
3
2
1

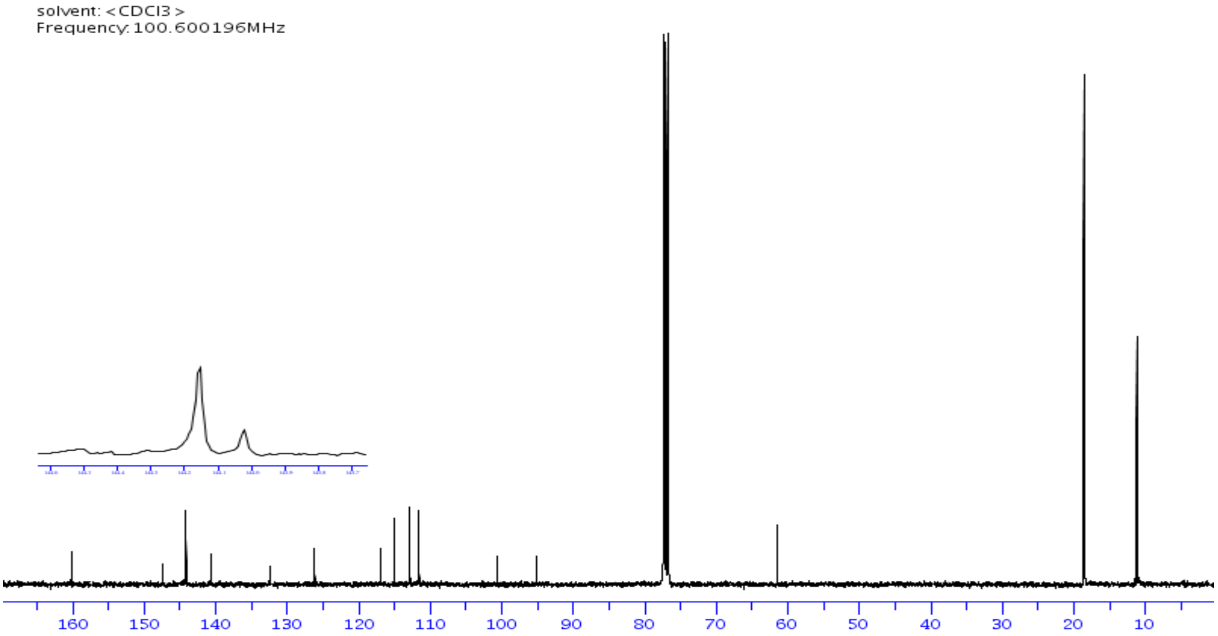
solvent: <CDCl3>
Frequency: 100.600196MHz
160
150
140
130
120
110
100
90
80
70
60
50
40
30
20
10

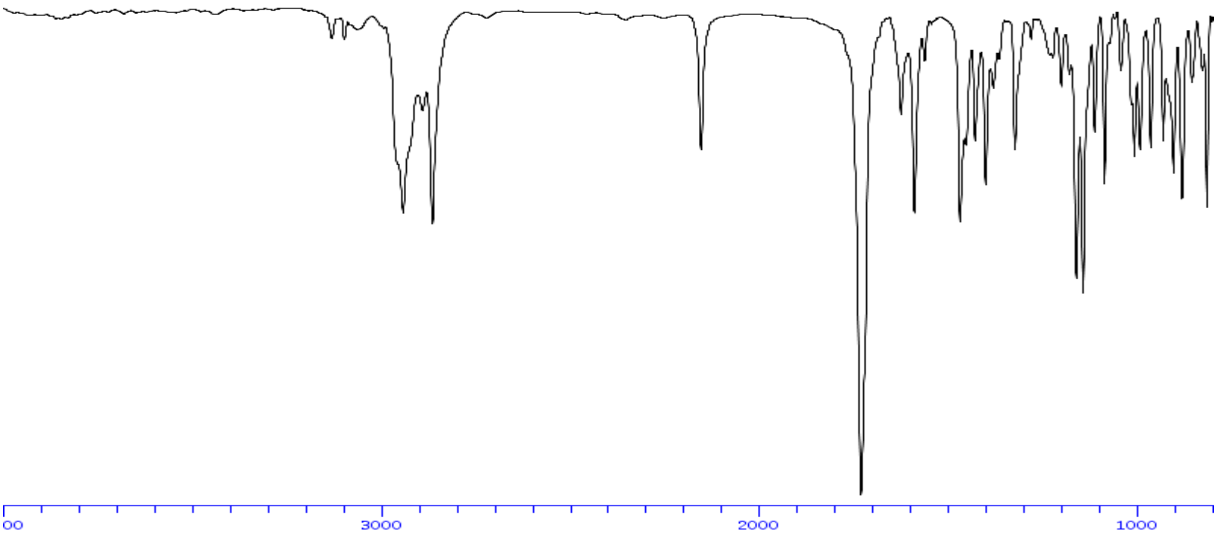
00
3000
2000
1000

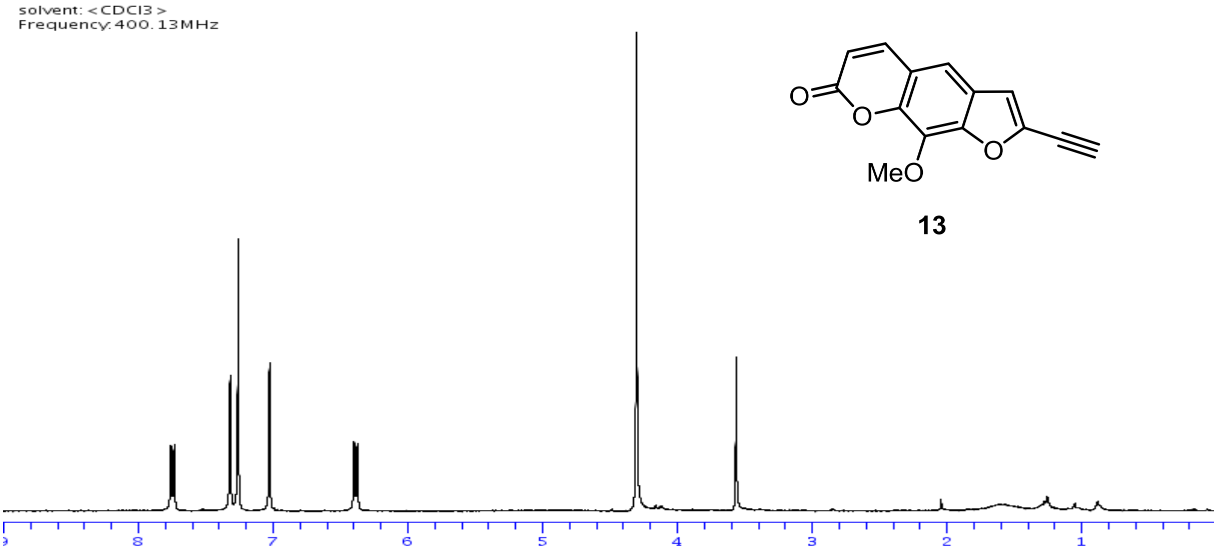
solvent: <CDCl3>
Frequency: 400.13MHz
O
O
MeO
O
13

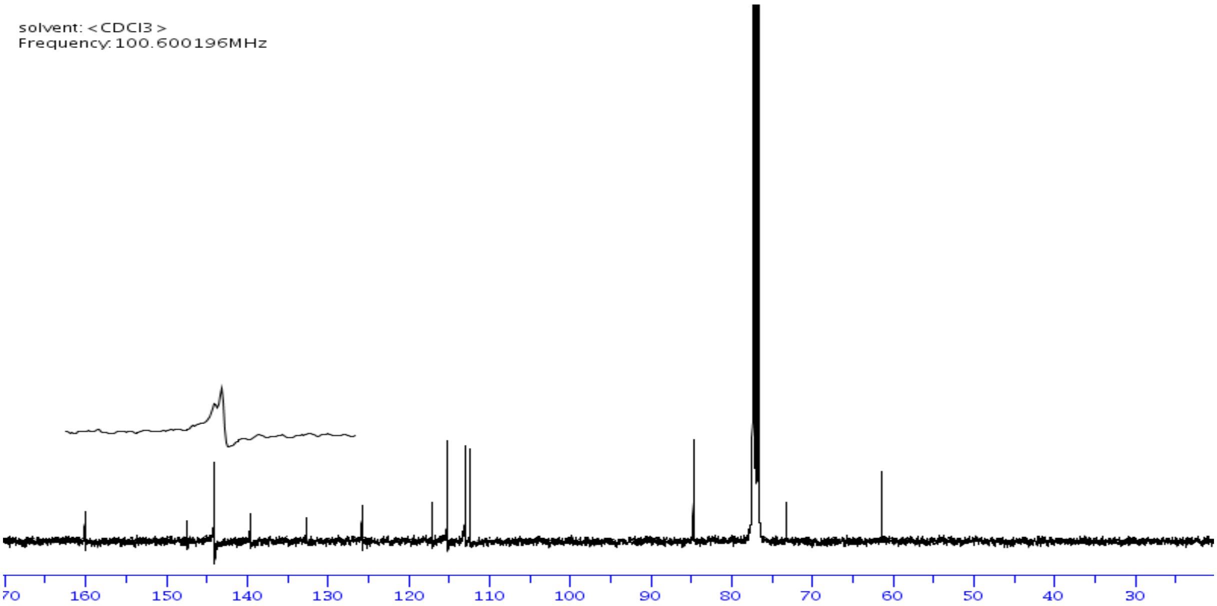
solvent: <CDCl3>
Frequency: 100.600196MHz

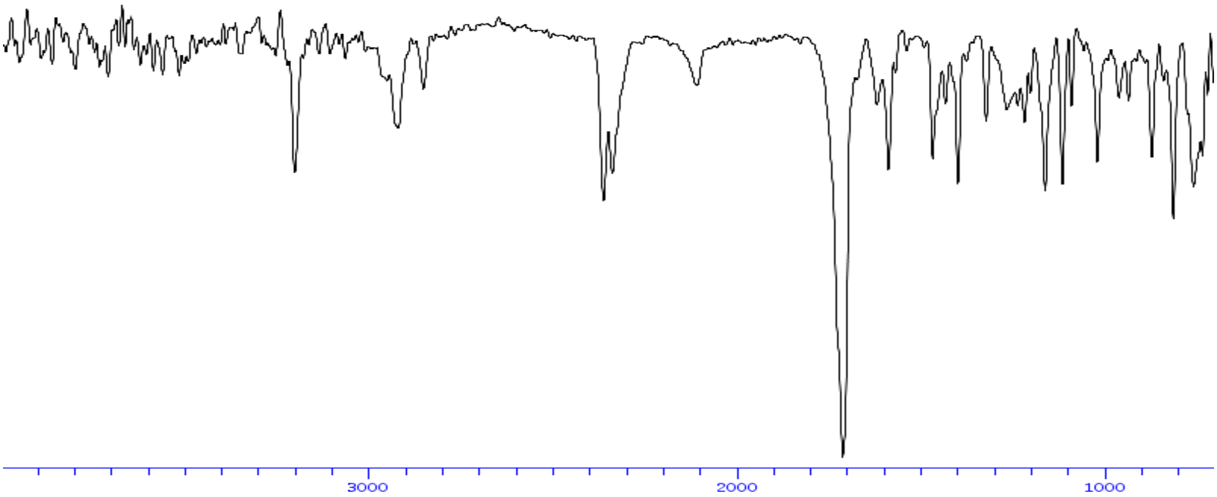

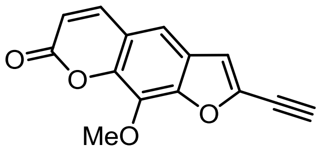
O
O
O
MeO

13

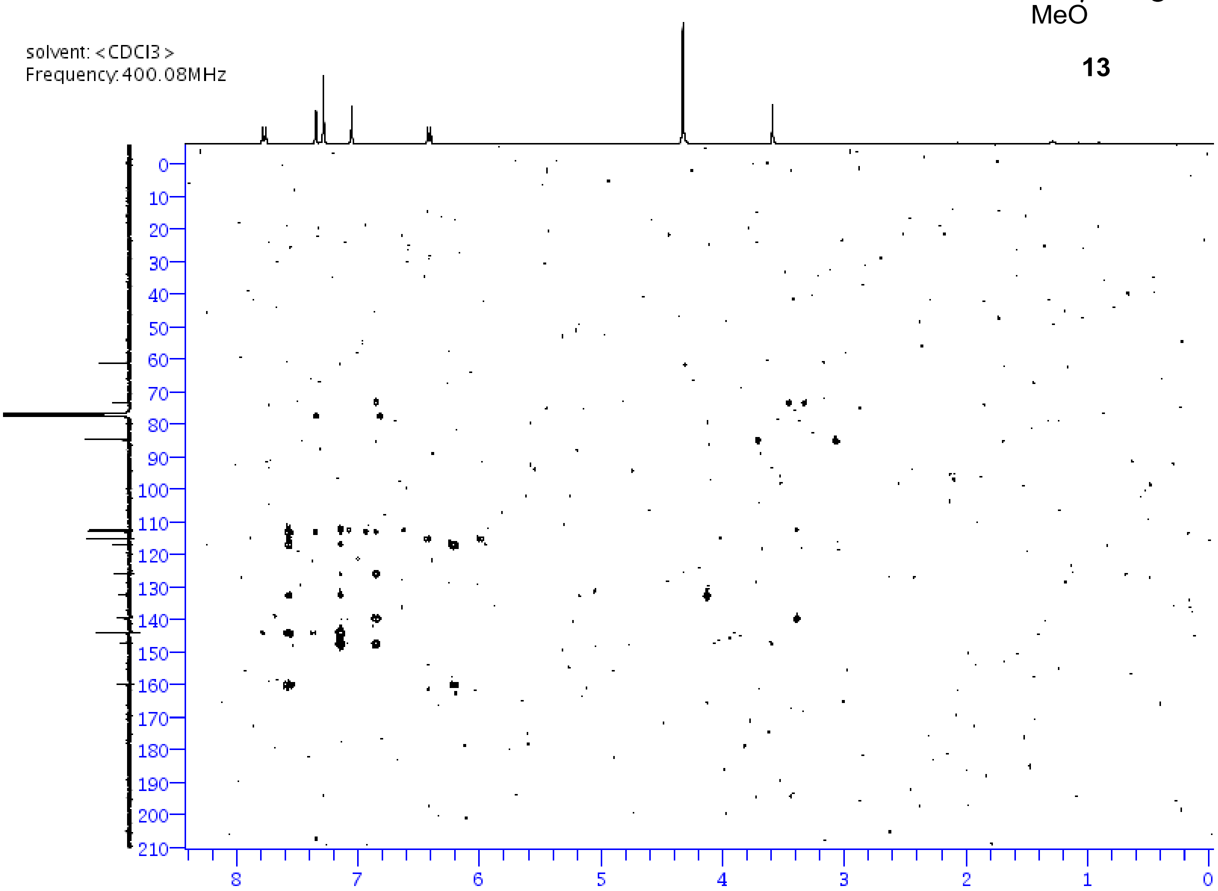
solvent: <CDCl3>
Frequency: 400.08MHz

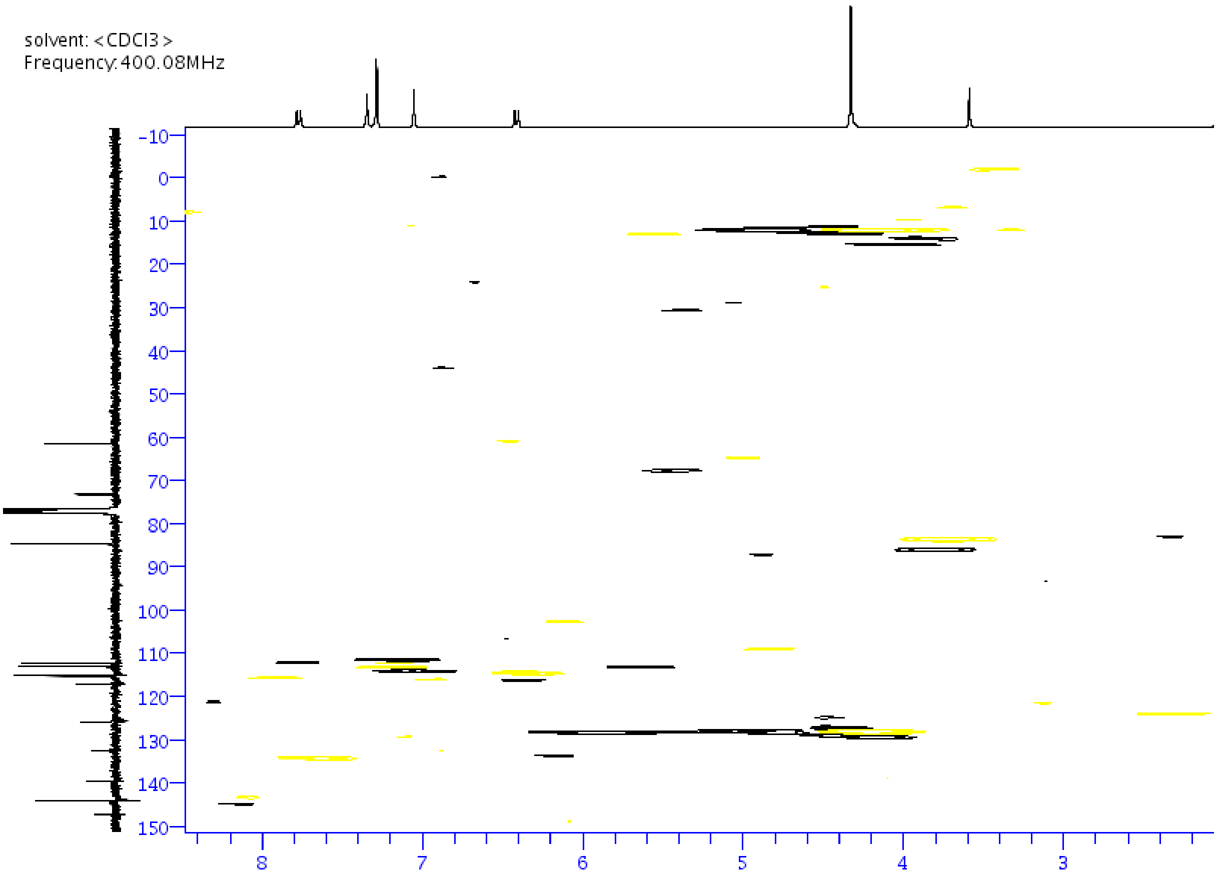
solvent: <CDCl3>
Frequency: 400.08MHz
